# Recent Progress on Layered Double Hydroxides and Their Derivatives for Electrocatalytic Water Splitting

**DOI:** 10.1002/advs.201800064

**Published:** 2018-05-23

**Authors:** Yanyong Wang, Dafeng Yan, Samir El Hankari, Yuqin Zou, Shuangyin Wang

**Affiliations:** ^1^ State Key Laboratory of Chem/Bio‐Sensing and Chemometrics Provincial Hunan Key Laboratory for Graphene Materials and Devices College of Chemistry and Chemical Engineering Hunan University Changsha 410082 P. R. China

**Keywords:** electrocatalysts, hydrogen evolution reaction, layered double hydroxides, oxygen evolution reaction, water splitting

## Abstract

Layered double hydroxide (LDH)‐based materials have attracted widespread attention in various applications due to their unique layered structure with high specific surface area and unique electron distribution, resulting in a good electrocatalytic performance. Moreover, the existence of multiple metal cations invests a flexible tunability in the host layers; the unique intercalation characteristics lead to flexible ion exchange and exfoliation. Thus, their electrocatalytic performance can be tuned by regulating the morphology, composition, intercalation ion, and exfoliation. However, the poor conductivity limits their electrocatalytic performance, which therefore has motivated researchers to combine them with conductive materials to improve their electrocatalytic performance. Another factor hampering their electrocatalytic activity is their large lateral size and the bulk thickness of LDHs. Introducing defects and tuning electronic structure in LDH‐based materials are considered to be effective strategies to increase the number of active sites and enhance their intrinsic activity. Given the unique advantages of LDH‐based materials, their derivatives have been also used as advanced electrocatalysts for water splitting. Here, recent progress on LDHs and their derivatives as advanced electrocatalysts for water splitting is summarized, current strategies for their designing are proposed, and significant challenges and perspectives of LDHs are discussed.

## 1 Introduction

Energy and environmental issues will be the crucial challenges in the 21st century, which are also problems for a global‐scale sustainable energy system.^1^, ^2^, ^3^, ^4^, ^5^ Fossil fuels, such as oil and coal, are nonrenewable resources. The utilization of fossil fuels has caused a number of environmental problems. Also, the increase in world population and the expansion of industrialization will lead to further growth of global energy demand from 18 TW in 2013 to 24 or 26 TW in 2040 according to the “new policies” or “current policies” scenarios.^2^ However, increasing demand for global energy will be accompanied with a fast rise in carbon dioxide emissions from 32 Gt year^−1^ in 2013 to 37 or 44 Gt year^−1^ in 2040. This has motivated and encouraged more people to pay close attention to the issue of energy supply, especially the climate change caused by the utilization of fossil fuel.^2^, ^6^ Thus, developing clean, renewable and affordable energy technologies is of critical importance to replace fossils fuels.

Due to the highest energy density and carbon‐free emission, hydrogen energy has received increasing attention and has been considered as one of the most critical candidate energies replacing fossils fuels.^7^, ^8^, ^9^, ^10^, ^11^, ^12^ Electrocatalytic splitting water is regarded as one of the most convenient and promising strategies to produce hydrogen.^13^ Hydrogen evolution reaction (HER) and oxygen evolution reaction (OER) are two important half reactions of splitting water, which are both vital for its overall efficiency.^14^, ^15^ The large overpotential and slow kinetic for both HER and OER impede seriously the practical applications of overall water splitting.^16^, ^17^, ^18^, ^19^ Accordingly, designing electrocatalysts with high electrocatalytic activity is extremely urgent and important for reducing the overpotential and improving the water‐splitting efficiency.

Precious metal (Pt) and noble‐metal oxides (RuO_2_ and IrO_2_) are recognized as excellent electrocatalysts for both HER and OER, respectively. However, high cost, limited resource, and poor operational stability impede their widespread commercial applications.^20^, ^21^, ^22^ Therefore, low‐cost, highly efficient, and stable long‐term electrocatalysts for water splitting are needed. Currently, transition‐metal (Fe, Co, Ni, Mn, and Mo)‐based catalysts including metal oxides,^23^, ^24^, ^25^, ^26^, ^27^, ^28^, ^29^, ^30^ hydroxides,^31^, ^32^, ^33^, ^34^, ^35^ phosphides,^36^, ^37^, ^38^, ^39^, ^40^, ^41^, ^42^ sulfides,^43^, ^44^, ^45^, ^46^, ^47^, ^48^ selenides,^49^, ^50^, ^51^, ^52^, ^53^, ^54^ and nitrides^55^, ^56^, ^57^, ^58^, ^59^, ^60^, ^61^, ^62^ have been highlighted as the most promising candidates of OER and HER electrocatalysts. Especially, layered double hydroxides (LDHs)^63^, ^64^, ^65^, ^66^, ^67^, ^68^, ^69^, ^70^, ^71^, ^72^, ^73^, ^74^, ^75^, ^76^, ^77^, ^78^, ^79^, ^80^, ^81^, ^82^, ^83^, ^84^, ^85^ and their derivatives (metal hydroxides, oxyhydroxides, oxides, bimetal nitrides, phosphides, sulfides, and selenides)^86^, ^87^, ^88^, ^89^, ^90^, ^91^, ^92^, ^93^, ^94^, ^95^, ^96^, ^97^, ^98^, ^99^, ^100^ have been widely investigated as electrocatalysts for water splitting owing to their availability, lower cost, and resource‐rich compared to the conventional noble metals and also their activity as well as their durability.

LDHs are a class of ion lamellar crystal mainly consisting of three parts: positively charged brucite‐like host layers, and the interlayer anions between host layers to balance charge and solvent molecules.^101^, ^102^, ^103^, ^104^, ^105^ LDHs are defined as the trivalent metal cations (e.g., Fe^3+^, Mn^3+^, Al^3+^, or Ga^3+^) that replace partial isomorphously bivalent metal cations (e.g., Co^2+^, Ni^2+^, and Fe^2+^) coordinated octahedrally by hydroxyl groups, forming the positively charged layer. The interlayer anions balance the additional positive charge of trivalent metal cations mainly containing inorganic or organic anion (e.g., CO_3_^2−^, NO_3_^−^, SO_4_^2−^, Cl^−^, and RCO_2_^−^).^104^, ^105^ Each hydroxyl in the LDH layer is oriented toward the intermediate layer region and may form hydrogen bonds with the interlayer anion and water molecules. Thus, the molecular formula can be expressed as [M^2+^_1−_ *_x_*M^3+^ *_x_*(OH)*_2_*][A*^n^* ^−^]*_x_*_/_ *_n_*·*z*H*_2_*O, where M^2+^ represents bivalent metal cations, M^3+^ represents trivalent metal cations, A*^n^* ^−^ is the interlayer anions, and *x* usually spans from 0.2 to 0.4.^106^ LDHs may also contain M^+^ and M^4+^. Generally, LDHs exhibit 2D layered nanosheets. This unique 2D lamellar structure endows LDHs with many advantages mainly i) the metal cations can be flexibly tunable in the host layers; ii) the interlayer anions and distance can also be changed by anion exchange method; iii) LDHs can also be exfoliated into ultrathin nanosheets by increasing the interlayer distance with the help of external force. Therefore, these aspects can efficiently regulate the physical and chemical properties of LDHs.

In addition, the unique lamellar and electronic structure endows LDHs with an average specific surface area and activity for electrocatalytic water splitting. Recently, considerable researches related to catalytic water splitting by using LDH‐based materials as catalysts have been published and showed a trend of extremely rapid growth.^104^, ^105^, ^106^ These studies have confirmed that LDH‐based electrocatalysts exhibited good electrocatalytic activities, especially for OER. However, these research results also further noted that inferior electrical conductivity, limited active sites, and intrinsically poor catalytic activity of active sites were identified as the vital factors for determining the electrocatalytic performance for water splitting.^104^, ^105^, ^106^ Thus, several typical strategies have been developed to improve the electrocatalytic activity of LDH‐based electrocatalysts. To enhance their electrical conductivity, LDH‐based electrocatalysts could be combined with conductive substrates^107^ (carbon nanotube, graphene, and carbon quantum dots) to improve their electrocatalytic performance.^101^ On the other hand, LDH electrocatalysts can be used as a precursor for synthesizing bimetallic nitrides, phosphides, selenides, and sulfides to improve their electrical conductivity.^94^, ^95^, ^96^, ^97^, ^98^, ^99^ In addition, increasing the number of active sites is another strategy to enhance the catalytic activities of LDH‐based electrocatalysts.^2^ Bulk LDHs with a large lateral size and thickness limit the exposure of active sites, leading to decreased electrocatalytic performance.^72^, ^73^, ^74^ Unique lamellar structure endows LDHs with flexible exfoliation feature which is helpful to form ultrathin LDH nanosheets with high specific surface area, exposing more active sites that boost the catalytic performance. At last, the intrinsic catalytic activity of active sites is an extremely vital factor to affect the performance of LDH‐based electrocatalysts.^2^ Surface engineering including heteroatom incorporation^66^, ^67^ and defect generation^72^, ^73^ on the 2D basal plane of LDH‐based catalysts can tune the electron distribution of adjacent atoms efficiently and further regulate and improve the intrinsic catalytic activity of active sites, resulting in a higher electrocatalytic performance.

Early reviews mainly focus on synthetic methods and techniques of LDHs, exfoliated technologies, and interlayer characteristics;^101^, ^102^ however, the application of the catalyst for water splitting is less involved, even if it is involved, it is very concise, not systematic, and the content is thin.^105^, ^106^ Apart from this, LDH derivatives are also a very good type of electrocatalyst for water splitting. However, there is no relevant summary in the early reviews. Compared to early reviews, the application of the LDHs catalyst in water decomposition is systematically and fully summarized in this review, mainly focusing on the design of electrocatalysts and the means of regulation. More importantly, this review involves various measures on improving the electrocatalytic properties of LDH‐based electrocatalysts, especially through the regulation and control of defect engineering. In addition, the content of LDH derivatives has also been added in this review, further enriching the content and providing ideas for the future design of the catalyst.

**Scheme 1:**
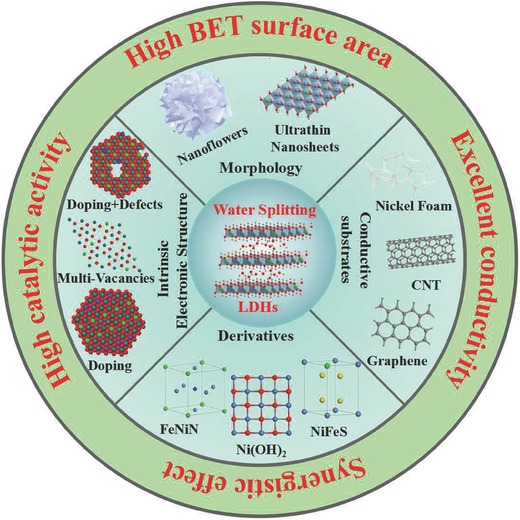
LDHs and their derivatives as electrocatalysts for water splitting.

In this review, we summarized the most recent advances on LDHs and their derivatives as electrocatalysts for water splitting (**Scheme** **1**, and **Tables** **1** and **2**). Notably, a series of LDH‐based catalysts, which can be obtained by different strategies including regulating morphology, constructing ultrathin nanosheets, and hierarchical nanostructures, combining conductive materials, exfoliation, heteroatom incorporation, and defect introduction, are emphatically reviewed in the following paragraphs. We also summarized the LDH derivatives as electrocatalysts for water splitting, including hydroxides, oxyhydroxides, oxides, bimetal nitrides, bimetal phosphides, bimetal sulfides, and bimetal selenides derived from LDH‐based materials. Intriguingly, what are considered to be the major barriers and challenges that need to be solved to improve the performance of LDH‐based catalyst in the field of electrocatalytic water splitting is how to increase their electrical conductivity, their number of active sites, and more importantly, to enhance the intrinsic catalytic activity of these active sites.

**Table 1:** Comparison of LDH‐based electrocatalysts

LDHs type	LDH‐based electrocatalysts	Electrolyte	Current density [mA cm^−2^]	Overpotential for OER or HER [mV]		Tafel slope [mV decade^−1^]	Ref.
Ultrathin nanosheets	NiFe LDHs	1 m KOH	10	≈350	OER	64	63
	NiV LDHs	1 m KOH	10	≈310	OER	50	63
	CoMn LDHs	1 m KOH	10	324	OER	43	64
Hierarchical nanostructures	NiFe‐LDH HMS	1 m KOH	10	239	OER	53	85
	3D NNCNTAs	0.1 m KOH	10	≈460	OER	65	121
	NiFe‐OH/NiFeP/NF	1 m KOH	10	199	OER	39	122
	Cu@NiFe LDHs	1 m KOH	10 10	199 116	OER HER	27.8 58.9	81
	NiFe‐LDH/NiCo_2_O_4_/NF	1 m KOH	50 10	350 257	OER HER	53 59	126
	NiCo_2_S_4_@NiFe‐LDH/NF	1 m KOH	60 10	201 200	OER HER	46.3 101.1	127
	FeOOH/NiFe‐LDH/NF	1 m KOH	10	208	OER	–	128
	NiFe:Pi/NiFe‐LDH/CFP	1 m KOH	10	290	OER	38	129
	CoSe/NiFe‐LDH/EG	1 m KOH	150 10	270 260	OER HER	57 –	130
Cation doping	NiFeCo LDHs	1 m KOH	10	220	OER	42	66
	NiFeMn LDHs	1 m KOH	20	289	OER	47	131
	NiCoFe LTHs/CFC	1 m KOH	10 10	239 200	OER HER	32 70	68
	La^3+^‐ and Ti^4+^‐doped NiFe LDHs	1 m KOH	10	260	OER	–	88
The anion in interlayer	MoO4^−^/NiFe LDHs	1 m KOH	10	280	OER	40	134
	PO_4_^3−^/NiFe LDH	1 m KOH	10	≈260	OER	≈42.1	69
	HPO_3_^2−^/NiFe LDH	1 m KOH	10	≈270	OER	≈40.6	69
	H_2_PO_2_^3−^/NiFe LDH	1 m KOH	10	≈240	OER	≈37.7	69
	CO_3_^2−^/NiFe LDH	1 m KOH	10	≈330	OER	≈44.3	69
Exfoliated and defective LDHs	Exfoliated NiFe LDHs	1 m KOH	10	300	OER	40	71
	Exfoliated NiCo LDH	1 m KOH	10	330	OER	41	71
	Exfoliated CoCo LDH	1 m KOH	10	350	OER	45	71
	Exfoliated NiCo LDH/CP	1 m KOH	10	300	OER	40	84
	H_2_O‐plasma exfoliated CoFe LDHs	1 m KOH	10	290	OER	36	72
	Ar‐CoFe LDHs	1 m KOH	10	266	OER	37.6	73
	Defect‐rich ultrathin CoFe LDHs	1 m KOH	10 10	300 255	OER HER	40 95	143
Combining LDHs with conductive substrate hybrids/composites	NiFe LDHs/NF	1 m NaOH	10 10	240 210	OER HER	––	107
	Ni_5_Fe LDH@NF	1 m KOH	10	210 133	OER HER	59 89	145
	NiFe LDHs/CNTs	0.1 m KOH 1 m KOH	5 5	≈290 ≈250	OER OER	35 31	75
	CNF/Fe‐doped Ni LDH	1 m KOH	10	220	OER	34	79
	NiFe‐rGO LDH hybrid	1 m KOH	10	206	OER	39	76
	nNiFe LDH/NGF	0.1 m KOH	10	337	OER	45	78
	CoAl LDH/3DGN	1 m KOH	10	252	OER	36	77
	NiFe LDH‐NS@DG hybrid	1 m KOH	10 20	210 115	OER HER	52 110	158
	CQD/NiFe LDH hybrid	1 m KOH	10	≈235	OER	30	161

**Table 2:** Comparison of LDH derivative electrocatalysts

Derivatives type	LDH derivative electrocatalysts	Electrolyte	Current density [mA cm^−2^]	Overpotential for OER or HER [mV]		Tafel slope [mV decade^−1^]	Ref.
Metal hydroxides, oxyhydroxides and oxides	Fe‐CoOOH/G	1 m KOH	10	330	OER	37	92
	Porous β‐Ni(OH)_2_	1 m KOH	20	236	OER	132	93
	Mono‐NiTi‐MMO	1 m KOH	10	320	OER	37	63
	NiFe‐MMO/CNT	1 m KOH	10	≈230	OER	≈45	87
Bimetal phosphides	Fe‐doped CoP	1 m KOH	10 10	230 78	OER HER	67 75	132
	(Fe*_x_*Ni_1−_ *_x_*)_2_P	1 m KOH	10	155	OER	66	167
	NiCoP	1 m KOH	10 10	280 32	OER HER	87 37	94
Bimetal nitrides	Ni_3_FeN nanoparticles	1 m KOH	10 10	280 158	OER HER	46 42	169
	FeNi_3_N/NF	1 m KOH	10 10	202 75	OER HER	40 98	98
	NSP‐Ni_3_FeN	1 m KOH	10 10	223 45	OER HER	40 75	91
	CoFeN	1 m KOH	20 10	222 22	OER HER	46 94	90
Bimetal sulfides	α‐FeNiS	0.5 m H_2_SO_4_	10	105	HER	40	95
Bimetal selenides	Ni*_x_*Fe_1−_ *_x_*Se_2_‐DO	1 m KOH	10	195	OER	28	96

## 2 Electrocatalytic Water Splitting

### 2.1 Electrocatalytic Kinetics and Important Parameters

In broad terms, the main role of electrocatalysts is to adsorb reactants and form intermediates on the surface of catalysts which accelerate the charge transfer between the electrode and the reactant.^2^, ^108^ Many electrocatalytic kinetic parameters can be used to evaluate the performance of electrocatalysts, among which the most critical parameters are overpotential (η) and Tafel slope (*b*).^108^ These two crucial parameters can even reveal the mechanism of electrocatalytic reaction to a certain extent. Some other parameters are also used to assess better and compare the activity of catalysts, including the total electrode activity, Faradaic efficiency, turnover frequency (TOF), as well as stability.^19^, ^109^, ^110^

#### 2.1.1 Overpotential (η)

Ideally, an electrocatalytic reaction can occur when the applied voltage is equal to the equilibrium potential. However, the electrocatalytic reaction can be carried out only when the applied potential is higher than the equilibrium potential to overcome the reaction barriers. According to the Nernst equation,^111^ for an electrocatalytic redox reaction, the applied potential can be formulated as Equation (1)

(1) $$E=E^{0}+RT/nF\;\mathrm{In}\;C_{O}/C_{R}$$

where *E* is the actually applied potential, *E* ^0^ is the standard potential, *R* is the universal gas constant, *T* is the absolute temperature, *n* is the number of transferred electrons in the reaction, *F* is Faraday constant, and *C*_O_ and *C*_R_ are the concentrations of oxidized and reduced reagents, respectively.

Overpotential (η) can be formulated as Equation (2)

(2) $$\eta =E-E_{\mathrm{eq}}$$

where *E*_eq_ is the equilibrium potential. The overpotential (η) reported in the literature is the overpotential at the current density of 10 mA cm^−2^.

#### 2.1.2 Tafel Equation and Tafel Slope (*b*)

In practical application, attaining a larger current density usually requires exerting a relatively large overpotential (η). In general, it is desirable only to require a smaller overpotential, meanwhile, obtain a faster growth of current density. The relationship of current density (*i*) and applied overpotential (η) can be expressed by the Butler–Volmer equation (Equation (3))^112^

(3) $$i=i_{0}\left[\exp\left(\alpha_{a}nFE/RT\right)+\exp\left(\alpha_{c}nF/RT\right)\right]$$

where *i*_0_ is the exchange current density, and α_a_ and α_c_ are the symmetrical coefficients for anode and cathode, respectively. Other parameters are the same as above.

At extremely high anode or cathode overpotential, Equation (3) can be simplified to Equations (4) and (5), respectively, which is the Tafel equation

(4) $$i\approx i_{0}\exp\left(\alpha_{a}nFE/RT\right)$$

(5) $$i\approx i_{0}\exp\left(\alpha_{c}nFE/RT\right)$$

To express the increase rates of current density with the increase of overpotential (η), the Tafel slope (*b*) was defined and expressed as Equation (6)

(6) b=∂η/∂ log i=2.303RT/αF

The smaller the Tafel slope (*b*) is, the faster the current density increases with the potential. Moreover, the value of Tafel slope (*b*) can speculate the rate‐limiting step of electrocatalytic reaction.

#### 2.1.3 The Total Electrode Activity

In the process of water splitting, the linear sweep voltammetry (LSV) or the cyclic voltammetry (CV) can reflect and evaluate the total electrode activity. Generally, the total current usually includes a non‐Faradaic capacitive current. Thus, it is only the estimated results of the total electrode activity by measuring LSV and CV results. Two different methods were employed to compare the total electrode activity. One method was used to compare the steady‐state current at a given overpotential and the other one is to compare the overpotential at a given steady‐state current density.

#### 2.1.4 Faradaic Efficiency

In an electrochemical system, the Faradaic efficiency usually represents the utilization efficiency of electrons taking part in reaction. For the OER, the Faradaic efficiency is defined as the molar ratio of the actually generated oxygen content (*n*_O2_) to the theoretically generated oxygen content (*n*_O2′_). Thus, the Faradaic efficiency for OER can be described as follows: Faradaic efficiency = *n*_O2_/*n*_O2′_ = 4*Fn*_O2_/(*It*), where *I* is a constant oxidation current, *t* is the active time at the constant oxidation current, and *F* is the Faraday constant. Similarly, the Faradaic efficiency for HER can be described as follows: Faradaic efficiency = *n*_H2_/*n*_H2′_ = 2*Fn*_H2_/(*It*).

#### 2.1.5 Turnover Frequency

TOF is usually utilized to evaluate the intrinsic catalytic activity of each catalytic site. The TOF value can be calculated by the following equation: TOF = (*jA*)/(4*Fn*), where *j* is the current density at a constant overpotential, *A* is the area of the working electrode, and *n* is the number of moles of the active materials. Notably, this TOF calculation method cannot give an accurate TOF value due to the different atom in catalyst with different catalytic activities. Nevertheless, it is still considered to be an efficient and important strategy to evaluate the electrocatalytic activity of similar electrocatalytic materials.

#### 2.1.6 Stability

In the practical application, the long‐term stability of catalysts is also a very important index to evaluate the quality of catalysts. Generally, two different test methods are used to assess the long‐term stability of catalysts. One method is to record the chronopotentionmetry curves at constant current densities of 10 mA cm^−2^ for a long period of time (>10 h) or the curves of time dependence of the current density under a constant overpotential (i.e., the *I*–*t* curve). The other method is to record and compare the LSV curves before and after a recycling CV (>2000 cycles).

### 2.2 The Reaction Mechanism of LDH‐Based Electrocatalysts

Electrocatalytic reaction is generally considered to be a surface reaction process. Hence, it is very vital to study the formation of intermediates during the process of electrocatalytic reactions and the reaction mechanism.^113^, ^114^ LDH‐based electrocatalysts are more attractive because of the unique lamellar structures, tunable compositions, and facile intercalation, resulting in excellent OER activities. More importantly, the mutual regulation of various metal components is remarkably beneficial for OER due to the optimal interaction between metal ions and oxygen intermediates. For example, Ni^2+^ and Fe^3+^ are mainly in NiFe LDHs at low potential.^113^ Further increasing the potential before the onset of OER, the Ni^2+^ was oxidized to Ni^3+^; however, the Fe cations remain as Fe^3+^. In other words, the NiFe LDHs was oxidized into γ‐Ni_1−_ *_x_*Fe*_x_*OOH (*x* < 25%) and NiFe LDH structure transformed into the Fe‐doped γ‐NiOOH structure leading to the shift for adsorption energies of oxygen intermediates.^113^ DFT+U calculation results further demonstrated that Fe‐doped LDHs changed the composition and structure of the oxidized catalyst.^113^ Meanwhile, DFT+U calculation of the OER overpotential explained the reason for the enhancement OER activity of Ni_1−_ *_x_*Fe*_x_*OOH with the increase of Fe content. Fe^3+^ in γ‐Ni_1−_ *_x_*Fe*_x_*OOH has a lower overpotential for OER compared with Ni^3+^ in either γ‐Ni_1−_ *_x_*Fe*_x_*OOH or γ‐NiOOH demonstrating the Fe sites in Ni_1−_ *_x_*Fe*_x_*OOH with higher catalytic activities.^113^ Recently, Müller and co‐workers also found that the catalytic process of NiFe LDHs involved an Fe(VI) intermediate.^114^ With the potential increase, the Ni^2+^ in NiFe LDHs was oxidation to Ni^3+^ and formed the NiOOH phase. Higher potential would further promote the formation of Fe^4+^. It was concluded that OER on NiFe LDHs is a two proton–one electron transfer, cf. the *cis*‐dioxo formation. They also found that the nickel‐hydroxide lattice played a stable, corrosion resistant role which could tightly bind catalytic Fe centers. Meanwhile, involving Ni(III)/(II) redox couple in OER mainly offered a reservoir of oxidizing equivalents and a conduit for transporting electrons to the electrode that is very helpful to water oxidation. The reactive *cis*‐dioxo–Fe(VI) fragment could only form in corner sites in the LDH lattice.^114^

Unique interlayer feature of LDH structure endows their unique physical and chemical properties that further influence their electrocatalytic properties. Tuning the interlayer distance of LDHs can efficiently improve electrochemically accessible surface areas of electrocatalysts and the diffusion of the reactants and products. More importantly, the types of intercalated anion usually have close relationship with the intrinsic catalytic activity of active sites for LDHs.^70^ Recently, Müller and coworkers found that the water oxidation activity of NiFe LDHs is related to the p*K*_a_ values of the conjugate acid of the intercalated anions.^70^ DFT calculations suggested that interlayer anion NO_3_^−^ in NiFe LDHs bound by its N atom to edge‐site iron is correlated with higher water oxidation activity.^70^

### 2.3 Catalytic Activity of Electrocatalysts

In general, the number and the intrinsic activity of active sites affect strongly the electrocatalytic activity of electrocatalysts for water splitting. Hence, two strategies can be used to improve the electrocatalytic activity efficiently. One strategy consists of increasing the number of active sites on a given electrode by increasing the loading or improving the catalyst structure to expose more active sites per gram.^2^, ^115^ Another strategy is to increase the intrinsic catalytic activity of each active site.^2^, ^115^ More importantly, combining these two strategies in one single system will improve the activity of electrocatalysts through synergetic effect and result in the significant improvements of electrocatalytic activity. As far as we know, for bulk LDH‐based electrocatalysts, the electrocatalytic activity is limited by the number and poor intrinsically activity of active sites because of the large particle size and thickness. Moreover, increasing the number of active sites of LDH‐based electrocatalysts can be realized by tuning the morphology, building hierarchical nanostructured catalytic system with a high surface area, and exfoliating bulk materials into ultrathin nanosheets. The intrinsic activity of electrocatalysts can be tuned efficiently via adjusting and regulating the material composition, mixed valence states of the compositional cations (redox couples), kinds of interlayered anion, metal–oxygen bond energy, oxygen vacancies, metal cations vacancies, electronic conductivity, and charge transfer capacity. In summary, the strategies for improving OER/HER electrocatalytic activity can be classified as i) choosing appropriate cations and anions for electrocatalysts, ii) optimizing the morphology of electrocatalysts, iii) improving the charge transfer process, iv) constructing composite or hybrid electrocatalysts, and v) introducing more edges or corner sites. Consequently, all these strategies usually play a synergetic and important role toward improvement of the electrocatalytic activity for HER/OER electrocatalysts.

## 3 Advanced LDH‐Based Electrocatalysts for Water Splitting

LDH‐based electrocatalysts have emerged as a promising candidate for OER catalysts in an alkaline medium owing to their environmental friendliness, earth abundance, thermal stability, and low cost. Also, the LDH‐based electrocatalysts have exhibited excellent electrocatalytic OER properties because of their unique 2D layered and electronic structure. However, limited active sites, poor intrinsic activity of active sites, and inferior electronic conductivity impeded the further improvement of electrocatalytic activity for LDHs. For these constraints, a series of efficient strategies were proposed as follows.

### 3.1 The Control of Morphology and Microstructure

Previous results have demonstrated that the morphology has a direct relationship with the exposure of active sites and the surface adsorption capability of the reactants, and these are crucial factor to influence the activity of electrocatalysts for water splitting.^116^, ^117^ The large particle size and thickness of the bulk LDHs limited the exposure of active sites and decreased the activity of active sites which significantly affected the electrocatalytic performance of LDHs for water splitting. To further increase the number of active sites and also improve their activity, the morphology of LDH‐based materials is tailored by synthesizing ultrathin nanosheets or constructing hierarchical nanostructure.

#### 3.1.1 Ultrathin Nanosheets

**Figure 1:**
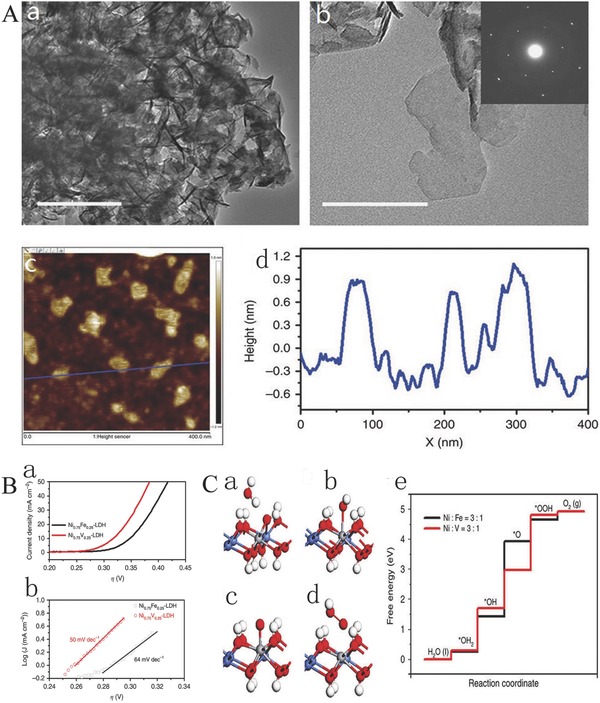
A) TEM and AFM. a,b) TEM images of Ni_0.75_V_0.25_‐LDH (inset of (b): selected area electron diffraction pattern); c) AFM image and d) height profile of Ni_0.75_V_0.25_‐LDH nanosheets. B) LSV curves and Tafel plots. a) LSV curves and b) Tafel plots of Ni_0.75_Fe_0.25_‐LDH and Ni_0.75_V_0.25_‐LDH. C) Adsorption geometries of the intermediates H_2_O, *OH, *O, and *OOH in (a)–(d), respectively. The red, blue, white, and gray atoms represent the O, Ni, H, and V atoms, respectively. The adsorption structures are similar to these when one Ni is substituted by Fe instead of V; e) the free‐energy landscape. Reproduced with permission.^63^ Copyright 2016, Nature Publishing Group.

Bulk LDHs usually faces severe challenges from sizeable lateral size and thickness. The thickness and size of LDHs strongly affect their physical and chemical properties, especially electrochemical properties by influencing the number of exposed active sites and their electronic properties. Reducing the size and thickness of LDHs could increase the specific surface area and also expose more active sites, in addition to the fabrication of more edge and corner sites, which all could contribute to an increased intrinsic catalytic activity of active sites, boost their electrical conductivity, increase the accessible sites, facilitate the permeation of electrolyte, and finally enhance the electrochemical activity for water splitting.^73^, ^83^, ^89^, ^118^ For instance, Sun and co‐workers synthesized monolayer nickel–vanadium layer double hydroxide (NiV LDHs) by a simple one‐step hydrothermal method.^63^ The NiV LDHs with a 3D morphology are assembled by ultrathin nanosheets (**Figure** **1**Aa,b) with a thickness of about 0.9 nm (Figure 1Ac,d). In this study, the unique monolayer NiV LDH nanosheets with high specific surface area facilitate the exposure of more active sites.^63^ The incorporation of vanadium is also helpful to improve the electrical conductivity of Ni(OH)_2_ and decreases the charge transfer resistance in OER process.^63^ The monolayer of NiV LDH nanosheets as OER electrocatalysts exhibits a high current density of 57 mA cm^−2^ at an overpotential of 350 mV for OER which was superior to NiFe LDH electrocatalysts for water oxidation (Figure 1B). To further understand the excellent catalytic activity of monolayer NiV LDH nanosheets, the mechanism for water oxidation using density functional theory (DFT) calculation was investigated (Figure 1C). DFT results demonstrate that the incorporation of vanadium in Ni(OH)_2_ nanosheets facilitates the formation and adsorption of OOH* (Figure 1Ce), indicating that NiV LDH nanosheets have a higher catalytic activity for water oxidation.^63^ These excellent catalytic properties can be mainly attributed to the increase in some active sites and the improvement of intrinsic catalytic activity of active sites.^63^

Also, Hu et al. also reported the synthesis of ultrathin CoMn LDH nanosheets as electrocatalysts for OER using a simple coprecipitation method at room temperature,^64^ and the new formed LDHs exhibit a platelet‐like structure with a diameter of 50–100 nm and an average thickness of 3.6 nm and show advanced electrochemical activity for OER with low overpotential of 324 mV at 10 mA cm^−2^ and small Tafel slope of 43 mV decade^−1^, which overmatches the Mn and Co oxides and hydroxides in 1 m KOH electrolyte.^64^ Moreover, After 12 h galvanostatic conditioning at an anodic current density of 10 mA cm^−2^, the ultrathin CoMn LDHs show ultralow overpotential of 293 mV at 10 mA cm^−2^. The improvement of OER is ascribed to the amorphous regions and active Co(IV) species on the surface, which result in a higher intrinsic catalytic activity of ultrathin CoMn LDH nanosheets for OER.^64^

#### 3.1.2 Hierarchical Nanostructures

The hierarchical nanostructure is an integrated architecture, which consists of nanoscale subunits, including 0D nanoparticles, 1D nanowires or nanotubes, and 2D nanosheets, in which these subunits are characteristically aligned in a well‐ordered fashion.^119^ Intriguingly, constructing and fabricating the hierarchical nanostructures can modulate the physical and chemical properties of original materials efficiently. This could also offer more opportunities to tune the physical and chemical performance for various technological applications.^119^

Previous studies have demonstrated that downsizing the nanomaterials could improve the surface to volume ratio in favor of exposing more active sites.^119^, ^120^ However, decreasing the size is always accompanied with increasing the charge transfer resistance. Therefore, rational design and fabrication of electrocatalysts with hierarchical nanostructures could effectively avoid this problem by providing seamless contact to the subunits. Meanwhile, the hierarchical nanostructures could avoid the aggregation, help the exposure of more active sites, and also accelerate the electrolyte penetration and diffusion, in addition to the fast release of bubbles at large current density profiting from the larger free space in electrocatalytic process.^119^, ^120^ Consequently, fabricating hierarchical nanostructures is a powerful strategy to improve the ultimate catalytic activity of the electrocatalysts.^120^ In this framework, Wei and co‐workers^85^ designed a 3D hierarchical NiFe‐LDH hollow microsphere (denoted as NiFe‐LDH HMS) for OER by in situ growth strategy using SiO_2_ as a sacrificial template, and the well‐defined LDH microspheres with a diameter of ≈350 nm, containing numerous highly distributed LDH nanoplatelets with a lateral size of ≈50 nm and thickness of ≈5 nm, were obtained. Importantly, Ni and Fe are uniformly distributed in the outer shell of microspheres. The 3D hierarchical NiFe‐LDH hollow microspheres exhibit a mesopore distribution (3–5 nm). Thus, benefiting from the 3D porous hierarchical structure, NiFe‐LDH HMS also show an excellent electrocatalytic activity for OER with an extremely small overpotential of 239 mV at 10 mA cm^−2^ and an extremely high current density of 71.69 mA cm^−2^ at a constant overpotential of 300 mV.^85^ This method of fabricating 3D hierarchical hollow microspheres has been extended to the synthesis of other LDH‐based materials.^85^

Jin and co‐workers reported a binary Ni–Co hydroxide‐based electrocatalyst with a unique sandwich‐like coaxial structure of the 3D Ni@[Ni^(2+/3+)^Co_2_(OH)_6–7_]*_x_* nanotube arrays (3D NNCNTAs).^121^ In this case, the Ni nanotube array with an open end is homogeneously coated with NiCo LDH nanosheets and is employed as a multifunctional interlayer to provide large surface area, fast electron transport, and support the outermost NiCo LDHs layer. These remarkable features have ensured excellent OER activity.^121^ Alshareef and co‐workers designed 3D hierarchical amorphous NiFe‐OH/NiFeP/NF as highly efficient and stable OER electrocatalysts.^122^ In their work, NiFe LDH nanosheet arrays are converted into bimetallic porous NiFeP nanosheet arrays using PH_3_ plasma treatment and subsequently constructed the 3D hierarchical amorphous NiFe‐OH/NiFeP nanosheet arrays. The NiFe‐OH/NiFeP was used for OER and exhibited outstanding electrocatalytic activity, mainly attributed to the decrease of the adsorption energy of H_2_O for NiFe‐OH/NiFeP in OER process because of the strong electronic interactions between NiFe‐OH and NiFeP and thus resulted in higher OER property. The advantages of the amorphous NiFe‐OH/NiFeP hierarchical structure with multilevel nanostructures mainly include i) the porous structure that facilitates the exposure of more active sites and improves the affinity contact with the electrolyte and the ion diffusion, ii) the hierarchical structure is convenient for the fast release of produced O_2_ bubbles under high current conditions, iii) the metallic NiFeP guarantees the fast electron transfer and decreases the charge‐transfer resistance, and iv) the amorphous NiFe‐OH nanosheets have an extremely high electrocatalytic activity for OER.

**Figure 2:**
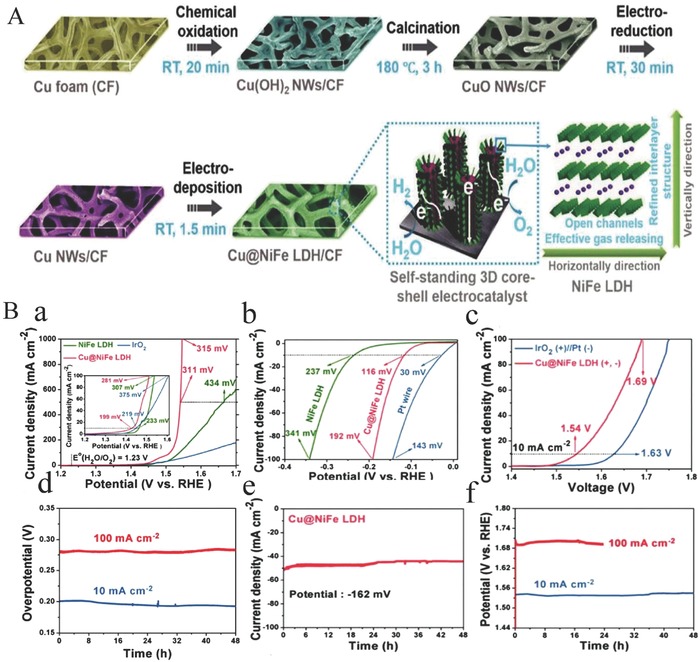
A) Schematic illustration of the fabrication procedures of the self‐standing 3D core–shell Cu@NiFe LDH electrocatalysts. B) Electrocatalytic performance and stability of 3D core–shell Cu@NiFe LDH electrocatalysts conducted in 1 m KOH. a) Polarization curves for OER; b) polarization curves for HER; c) polarization curves for overall water splitting; d) chronopotentiometry curves for OER at constant current densities of 10 and 100 mA cm^−2^; e) time dependence of the current density for HER under a constant overpotential of 162 mV to afford a current density of 50 mA cm^−2^; f) chronopotentiometry curves for overall water splitting at constant current densities of 10 and 100 mA cm^−2^. Reproduced with permission.^81^ Copyright 2017, The Royal Society of Chemistry.

Amorphous materials were shown to exhibit higher electrocatalytic activity than the crystalline catalysts.^123^, ^124^, ^125^ Ren and co‐workers reported a facile and scalable approach to fabricate efficient 3D bulk catalysts of core–shell nanostructures, in which few‐layer NiFe LDH nanosheets are grown on Cu nanowire core supported on Cu foams, toward overall water splitting, as shown in **Figure** **2**A.^81^ This catalyst possesses a unique 3D core–shell nanostructure, super large specific area, and abundant active sites. Moreover, the excellent conductivity of Cu nanowires facilitates the transfer of electrons from Cu nanowires to NiFe LDH nanosheets. In addition, NiFe LDH has a unique layered structure, and the gap between layers provides an open channel for the rapid release of gas molecules, providing a guarantee for the excellent performance of electrodes at high current densities. Therefore, in the alkaline electrolyte, this 3D core–shell nanostructure electrode exhibits excellent electrocatalytic activity and stability for both OER and HER (Figure 2Ba,b). The performance is better than the Pt/IrO_2_ electrocatalysts for overall water‐splitting system (Figure 2Bc). Also, this electrode displays excellent stability for both OER and HER (Figure 2Bd,e), and for overall water‐splitting reaction (Figure 2Bf). The preparation method is simple, has the potential of low energy consumption, and does not produce pollution, which is suitable for preparing large size sample and has a good application prospect.

Besides, other 3D hierarchical structures of LDH‐based materials including NiFe‐LDH/NiCo_2_O_4_/NF,^126^ NiCo_2_S_4_@NiFe‐LDH/NF,^127^ FeOOH/NiFe‐LDH/NF,^128^ NiFe:Pi/NiFe‐LDH/CFP,^129^ and CoSe/NiFe‐LDH/EG^130^ were synthesized as OER or HER electrocatalysts with excellent electrocatalytic activity and good durability.

### 3.2 Cationic Doping

The versatile composition of LDHs allows wide selection of earth‐abundant elements and endows them with unique catalytic activities when active components are suitably located in the structure, making them particularly promising OER catalysts. Compared to LDH/oxides, layered triple hydroxides/oxides exhibited a better electrocatalytic activity that may be due to the doping of a third metal in LDHs, which efficiently tunes the morphology, the electronic structures, and electrical conductivity of electrocatalysts.^66^, ^67^, ^68^ Therefore, cationic doping is considered to be an efficient method to improve the activity of electrocatalysts.

Recently, Yang and co‐workers reported a ultrathin FeNiCo LDH nanosheet through Co intake mediating the formation of ultrathin nanosheets.^66^ In this work, trimetal FeNiCo LDH (FeNi_9_Co LDHs and FeNi_8_Co_2_ LDHs) ultrathin nanosheets with atomic thickness were prepared using the one‐step hydrothermal method. It is found that while increasing the Co content in the precursor solution, the thickness of as‐synthesized LDH nanosheets decreases from FeNi_10_ LDH nanosheets of ≈2 nm to FeNi_9_Co LDH nanosheets of 1.5–1.8 nm to FeNi_8_Co_2_ LDH nanosheets of 1.2–1.5 nm. The specific surface area increases from 46.05 m^2^ g^−1^ for FeNi_9_Co LDH nanosheets to 54.18 g^−1^ for FeNi_10_ LDH nanosheets to 80.44 g^−1^ for FeNi_8_Co_2_ LDH nanosheets. Importantly, decreasing the thickness and increasing the surface area might expose more catalytically active sites. Moreover, the incorporation of Co leads to an i) improved conductivity of FeNi_10_ LDH nanosheets, ii) decreased charge transfer resistance affinity related to a fast reaction rate and iii) enhanced electrocatalytic performances of catalysts. The insertion of Co also modulates the electronic structure of the active sites, which greatly improves the intrinsic activity of active sites.^66^

**Figure 3:**
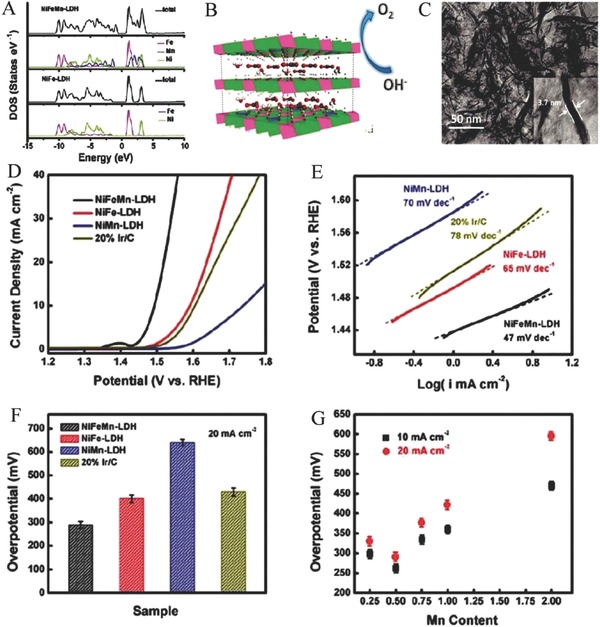
A) Total DOS and partial DOS () curves of NiFeMn‐LDH and NiFe‐LDH. B) Structural image of NiFeMn‐LDH; C) TEM image of NiFeMn‐LDH; D) LSV curves of NiFeMn‐LDH, NiFe‐LDH, NiMn‐LDH, and the commercial Ir/C catalyst; E) the corresponding Tafel plots; F) the corresponding overpotential at a current density of 20 mA cm^−2^; G) the required overpotential of the different Mn content in the ternary LDHs at 10 and 20 mA cm^−2^, respectively. Reproduced with permission.^131^ Copyright 2016, The Royal Society of Chemistry.

Importantly, DFT calculation results prove that Mn^4+^ doped into NiFe LDHs could narrow the bandgap improving the electric conductivity of NiFe LDHs (**Figure** **3**A). Hence, Duan group's designed Mn^4+^‐doped NiFe LDHs, namely NiFeMn LDHs, via a simple coprecipitation method at room temperature.^131^ The NiFeMn LDHs have a flower‐like structure assembled by the ternary LDH nanosheets, an average lateral size of ≈50 nm, and a thickness of ≈3.7 nm (Figure 3B,C), and show excellent OER performance with the lowest onset potential of 200 mV and fastest OER current increase compared to the undoped NiFe LDHs and NiMn LDHs (Figure 3D). Moreover, a fast kinetic process with low Tafel slope of 47 mV decade^−1^ is observed (Figure 3E). To achieve 20 mA cm^2^, the new prepared LDHs only need an overpotential of ≈289 mV which is less than 401 mV of NiFe LDHs and 640 mV of NiMn LDHs (Figure 3F). The optimized transition metal ratio is 3:1:0.5 for Ni, Fe, and Mn (Figure 3G). The DFT results show that NiFe LDHs have a bandgap of about 2.1 eV between the valence bands and conduction bands. However, Mn^4+^ doped into NiFe LDHs results in a decrease of the bandgap, suggesting the presence of more conductive electronic structure. The sheet resistance of NiFeMn LDHs disk‐shaped pellet is 1.6 × 10^3^ Ω square^−1^ which is lower than that of the NiFe LDH of 2.2 × 10^3^ Ω square^−1^, confirming the higher conductivity of NiFeMn LDH. Doping Mn into NiFe LDHs also facilitates the adsorption of the *O and *OH intermediates, accelerating the OER process. Therefore, the superior OER properties of NiFeMn LDH could mainly ascribed to the tunable electronic structure and enhanced electric conductivity, resulted from the incorporation of Mn^4+^.^131^

Li and co‐workers also reported the synthesis of NiCoFe layered triple hydroxides with a porous structure supported on carbon fiber cloth (NiCoFe LTHs/CFC) using the electrodeposition method. The as‐synthesized NiCoFe LTHs/CFC has the structure of porous networks assembled by the interconnected nanosheets,^68^ which guarantee a fast transportation and facilitate the diffusion of reactants and products. In this study, the introduction of CFC accelerates the electron transport and also decreases the resistance of NiCoFe LTHs/CFC, which are found to benefit the overall water splitting. Indeed, outstanding OER activity with a low overpotential of 239 mV at 10 mA cm^−2^ and good HER performance was observed, in addition to the low initial potential of 1.51 V and the low potential of 1.55 V at 10 mA cm^−2^.

Furthermore, elemental doping has also been considered as an efficient strategy for tuning the coordination valence and surface chemical environment of electrocatalysts.^132^, ^133^ Recently, Jin and co‐workers reported an Al element–doped ultrathin Ni_3_FeAl*_x_*‐LDH nanosheets as the OER electrocatalyst.^67^ The ultrathin Ni_3_FeAl*_x_*‐LDH nanosheets significantly improve the OER activity compared with that of Ni_3_Fe‐LDH, this could be attributed presumably to increased number of low‐coordinated Ni and Fe atoms after incorporating the trivalent Al ions in the NiFe LDHs. Moreover, the partial etching of Al^3+^ species on the Ni_3_FeAl*_x_*‐LDH nanosheets surface using some corrosive chemicals would further result in the exposure of more active sites. More interestingly, the content of incorporated Al strongly affects the formation and the atomic ratio of Ni^3+^ and the electrocatalytic activity of Ni_3_FeAl*_x_*‐LDH nanosheets, indicating that the increase of Ni^3+^ concentration in Ni_3_FeAl*_x_*‐LDH would greatly improve the electrocatalytic activity of active sites and lead to significant enhancement of OER performance.^67^ Müller and co‐workers synthesized surfactant‐free mixed‐metal LDHs as water oxidation nanocatalysts by pulsed‐laser ablation in liquids. Doping with La^3+^ and Ti^4+^ into NiFe LDHs further enhanced electrocatalytic activities with a low overpotential of 260 mV at 10 mA cm^−2^.^88^

In summary, cationic doping in LDH‐based electrocatalysts is a critical strategy for enhancing their catalytic activities, which can be attributed to the following reasons: i) cationic doping could change the morphology of LDHs, decrease the thickness and size of LDH nanosheets which increases the surface area and exposes more active sites; ii) this could also improve the electrical conductivity of LDHs, accelerating thus the kinetic process of OER; and iii) the doped cationic species could act as the active sites, to tune the electronic structure of catalyst surface and also affect the surface chemical environment (the coordination number of surrounding active atoms and the valence of element) of electrocatalysts and finally improve the intrinsic electrocatalytic activity of active sites for LDHs. Therefore, combining the aforementioned advantages significantly improves the electrocatalytic activity and performance of LDHs after cationic doping. Moreover, this strategy can be extended to other metal‐based electrocatalysts.

### 3.3 Tuning the Anion and Spacing in Interlayer

**Figure 4:**
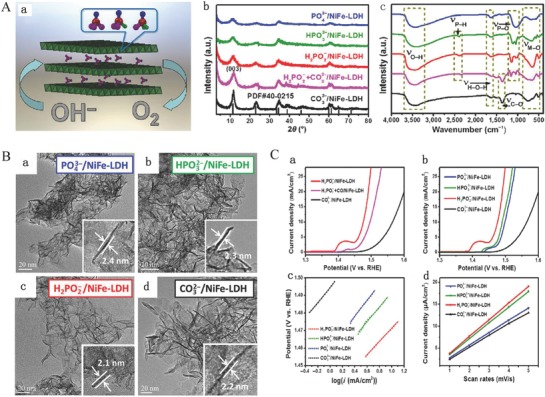
A) Structural scheme of phosphate, phosphite, and hypophosphite‐intercalated NiFe‐LDH a); b) the XRD patterns for standard NiFe‐LDH and as‐synthesized CO_3_^2−^/NiFe‐LDH, H_2_PO_2_^−^+CO_3_^2−^/NiFe‐LDH, H_2_PO_2_^−^/NiFe‐LDH, HPO_3_^2−^/ NiFe‐LDH, and PO_4_^3−^/NiFe‐LDH; c) FTIR spectra of the all as‐synthesized NiFe‐LDH nanosheets. B) TEM images of a) PO_4_^3−^/NiFe‐LDH, b) HPO_3_^2−^/ NiFe‐LDH, c) H_2_PO_2_^−^/NiFe‐LDH, and d) CO_3_^2−^/NiFe‐LDH; insets show the representative thickness of the corresponding LDH. C) Electrocatalytic performance of as‐synthesized samples: a) polarization curves of H_2_PO_2_^−^/NiFe‐LDH, H_2_PO_2_^−^+CO_3_^2−^/NiFe‐LDH, and CO_3_^2−^/NiFe‐LDH; b) polarization curves of PO_4_^3−^/NiFe‐LDH, HPO_3_^2−^/NiFe‐ LDH, H_2_PO2^−^/NiFe‐LDH, and CO_3_^2−^/NiFe‐LDH; c) corresponding Tafel plots; d) *C*_dl_ calculations for the four catalysts. Reproduced with permission.^69^ Copyright 2017, Springer Link.

**Figure 5:**
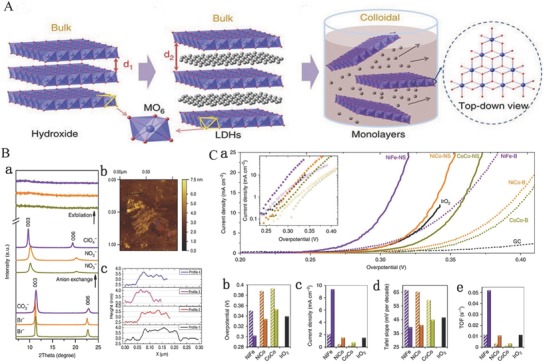
A) Schematic diagram of exfoliating LDH. a) LDHs; b) LDHs with interlayer anions and water molecules; *d*_2_ > *d*_1_ (interlayer distance); c) exfoliated LDHs into monolayers nanosheets. B) XRD patterns and AFM images. a) XRD patterns including NiFe LDH, NiCo LDH, and CoCo LDH; b) AFM image of monolayer nanosheets of NiCo LDH; c) the corresponding height profile of monolayer nanosheets of NiCo LDH. C) Electrochemical properties. a) LSV polarization curves of synthesized LDHs and IrO_2_ at 5 mV s^−1^ in 1 m KOH electrolyte; b) overpotential of synthesized LDHs and IrO_2_ at 10 mA cm^−2^; c) current densities of synthesized LDHs and IrO_2_ at an overpotential of 300 mV; d) corresponding Tafel slopes; e) TOF calculated at an overpotential of 300 mV. Reproduced with permission.^71^ Copyright 2014, Nature Publishing Group.

LDHs are a family of 2D layered materials built from the alternate arrangement of brucite‐like cationic layers and charge‐balancing anions in the interlayer region. The interlayer anion is also an important factor that influences the electrocatalytic performance of electrocatalysts that mainly involves two aspects: on the one hand, the interlayer distance of intercalation anion may increase electrochemically accessible surface areas of LDHs and accelerate the diffusion of the reactants and products; on the other hand, the types of intercalated anion may affect the intrinsic catalytic activity of active sites for LDHs.^69^, ^70^ Recently, NiFe LDH nanosheets with different interlayer anions were reported.^70^ In this study, the influence of intercalated anions on OER properties was investigated and found that the water oxidation activity of NiFe LDHs strongly depends on the p*K*_a_ values of the conjugate acid of the intercalated anions. Li and co‐workers reported an ultrathin NiFe LDHs intercalated with molybdate anions as OER electrocatalysts by the one‐step hydrothermal method. These OER electrocatalysts with structure nanosheets exhibited a texture being similar to dried graphene oxide, which had a lateral size of about several hundred nanometers. The thickness of 2–3 atomic layers and the interlayer distance of ≈0.7 nm confirmed the intercalation of molybdate anions.^134^ These electrocatalysts were served as efficient and durable OER electrocatalysts in 1 m KOH, which need a low overpotential of 280 mV at 10 mA cm^−2^ outperforming the Ir/C and typical NiFe LDH nanosheets.^134^ Guan and co‐workers also reported different interlayer distances (0.78 and 0.95 nm, respectively) of NiFe LDH nanosheets by an in situ intercalation method, the formamide as the intercalation anion, to expand the intercalated distance of electrodeposited NiFe LDH electrode.^135^ Interestingly, increasing the interlayer spacing for NiFe LDH nanosheets improves the OER activity.^135^ Recently, Sun and co‐workers also reported the preparation of phosphorus oxoanion including phosphate, phosphite, and hypophosphite anion intercalated NiFe LDH electrocatalysts (denoted as PO_4_^3−^/NiFe‐LDH, HPO_3_^2−^/NiFe‐LDH, and H_2_PO_2_^−^/NiFe‐LDH) for OER via a coprecipitation method (**Figure** **4** Aa).^69^ The X‐ray diffraction (XRD) patterns and Fourier transform infrared spectroscopy (FTIR) spectra confirmed that all phosphorus oxoanion intercalated NiFe LDH have been successful synthesized (Figure 4Ab,c). Transmission electron microscopy (TEM) images demonstrate that all the as‐synthesized samples exhibit a lateral size of ≈50 nm and a thickness of ≈2 nm (2–3 atomic thickness) as shown in Figure 5Ba–d. Their electrocatalytic activity is evaluated for OER in 1 m KOH electrolyte (Figure 4C). The results show that increasing the interlayered H_2_PO_2_^−^ anion concentration for typical CO_3_^2−^/NiFe‐LDH nanosheets further improves the OER properties (Figure 4Ca). The H_2_PO_2_^−^/NiFe‐LDH nanosheets have the earliest onset potential of ≈1.45 V compared with CO_3_^2−^/NiFe‐LDH, PO_4_^3−^/NiFe‐LDH, and HPO_3_^2−^/NiFe‐LDH nanosheets (Figure 4Cb). For H_2_PO_2_^−^/NiFe‐LDH nanosheets, the curve of Ni^2+^/Ni^3+^ oxidation greatly shifted toward lower potential compared with that of other samples (Figure 4Cb), implying the change of the electronic structure surrounding the Ni sites. Besides, the H_2_PO_2_^−^/NiFe‐LDH nanosheets exhibit a faster kinetic process with the lowest Tafel slope of ≈37.7 mV decade^−1^ in contrast to that of CO_3_^2−^/NiFe‐LDH with ≈44.3 mV decade^−1^, PO_4_^3−^/NiFe‐LDH with ≈42.1 mV decade^−1^, and HPO_3_^2−^/NiFe‐LDH with 40.6 mV decade^−1^ (Figure 4Cc). Meanwhile, the H_2_PO_2_^−^/NiFe‐LDH nanosheets also show larger electrochemical active surface area (ECSA) demonstrating that the H_2_PO_2_^−^/NiFe‐LDH nanosheets have a higher intrinsic activity for OER (Figure 4Cd).^69^

In summary, given the unique 2D layered structure of LDHs with tunable interlayered spacing via different intercalated anions, the improvements of OER properties can be mainly attributed to increased electrochemically accessible surface area, fast diffusion of the reactants and products in OER process, and enhanced intrinsic catalytic activity of active sites for LDH electrocatalysts. This method can also be extended to other similar 2D layered materials.

### 3.4 Exfoliation of LDHs and Tuning Electronic Structure and Introducing Defects

Most bulk LDHs usually suffer from a limited specific surface area and poor conductivity, which severely affect the catalytic activities of LDHs. Previous investigations have demonstrated that 2D nanomaterials with single or few‐atomic layer not only dramatically increase the surface area to facilitate the exposure of more active sites, but also extremely benefit the enhancement of chemical and physical relativities. Moreover, the exfoliation of bulk materials to ultrathin nanomaterials is invariably accompanied with the formation of numerous edges and corner sites together with the appearance of more dangling bonds resulted in the active sites with lower coordination.^136^, ^137^, ^138^, ^139^ As far as we know, the intrinsically catalytic activity of per active sites has a direct influence on the catalytic activities. Moreover, It is widely believed that the edge and corner sites of the catalysts exhibit higher catalytic activities compared with the basal planes sites due to the more dangling bonds and the decreased coordination number of active sites which are facilitated for the adsorption of active intermediates.^140^, ^141^ This also suggests that the coordination number plays a vital role in influencing the catalytic performance so that the characterization of coordination number is necessary for an in‐depth understanding of the catalysis. Recently, liquid exfoliation of layered materials has emerged as a transformative process in producing novel 2D materials with drastically improved surface activity compared to their bulk counterparts, especially LDH‐based materials. Song and Hu synthesized single‐layer NiFe LDHs, NiCo LDHs, and CoCo LDH nanosheets by liquid exfoliation of bulk LDHs (**Figure** **5**A).^71^ First, the interlayer distance of bulk LDHs was increased by anion exchange (the anions of ClO_4_^−^ were used to exchange CO_3_^2−^ of NiFe LDHs and the NO_3_^−^ for CoCo and NiCo LDHs) to allow the processing of bulk LDHs into singer‐layer nanosheets. Subsequently, the samples after anions exchange bulk NiFe, NiCo, and CoCo LDHs were dispersed in purged formamide and stirred for 24 h. Finally, the suspensions of bulk LDHs became a clear solution. XRD and atomic force microscopy (AFM) results confirm that the bulk LDHs are successfully exfoliated into single‐layer LDH nanosheets (Figure 5Ba–c). The OER properties of bulk and single‐layer LDH nanosheets were further studied and showed that the OER properties of single‐layer LDH nanosheets were far beyond bulk LDHs, including the NiFe, NiCo, or CoCo LDHs (Figure 5Ca–d). Meanwhile, all single‐layer LDH nanosheets exhibit a lower Tafel slope (Figure 5Cc) and a higher TOF value (Figure 5Cd) confirming that single‐layer nanosheets have higher electron conductivity and a faster electron transport than bulk particles. More in‐depth studies show that the significant higher electrocatalytic activity of single‐layer LDH nanosheets than that of bulk LDHs is attributed to both the increase in active site density and conductivity. Moreover, the edge sites also might act as an active site with higher electrocatalytic activity for water oxides, and the increase of edges sites originated from the reduction in size might result in significant improvement of OER properties for single‐layer LDH nanosheets.^71^

**Figure 6:**
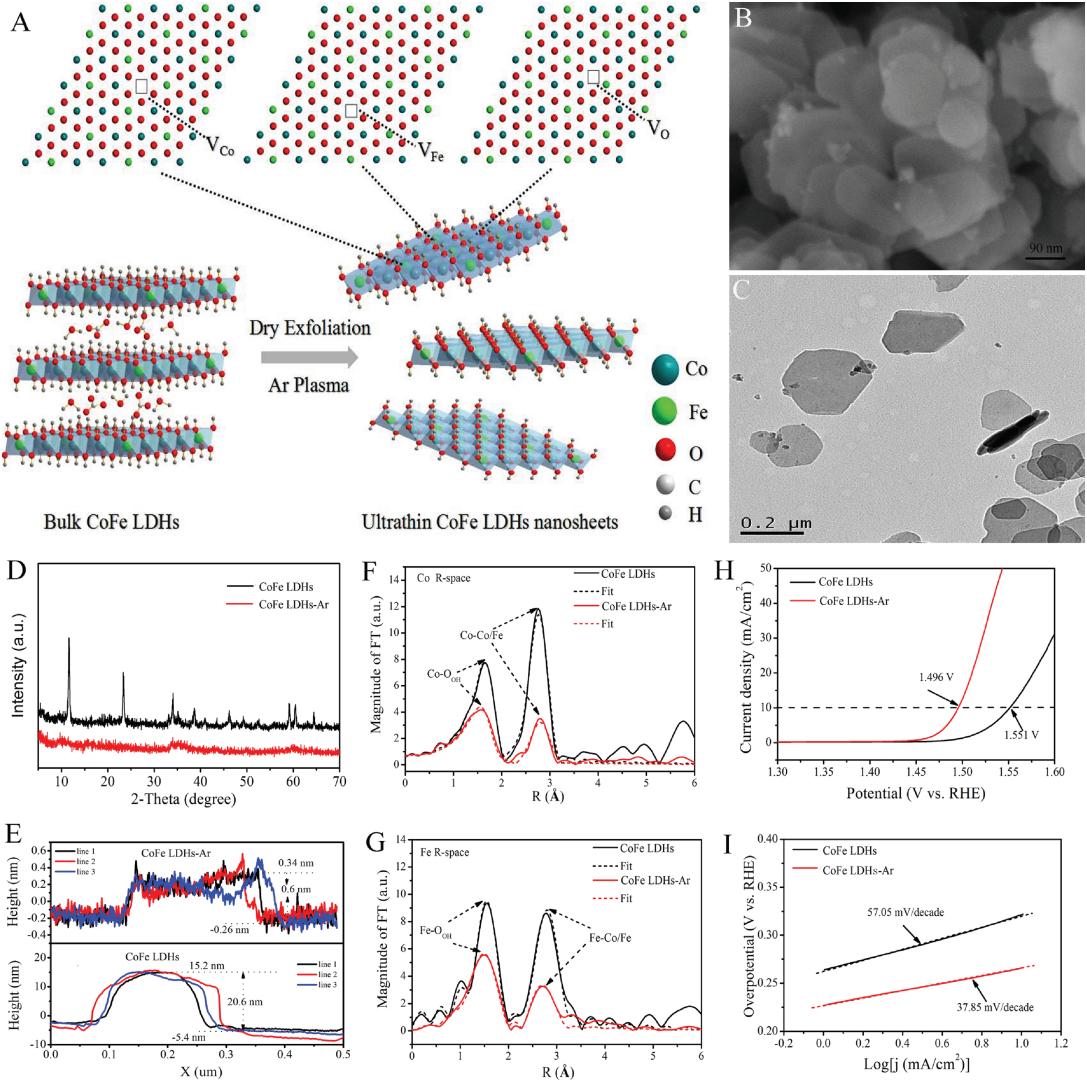
A) Illustration of Ar plasma exfoliated CoFe LDH nanosheets; B) SEM and C) TEM image of the ultrathin CoFe LDHs‐Ar nanosheets; D) XRD patterns of the bulk CoFe LDH nanosheets and ultrathin CoFe LDHs‐Ar nanosheets; E) the corresponding height curves; F) magnitude of *k* ^3^‐weighted Fourier transforms of the Co edge XANES spectra for bulk‐CoFe LDHs and ultrathin CoFe LDHs‐Ar with the corresponding curve‐fitting results; G) magnitude of *k* ^3^‐weighted Fourier transforms of the Fe edge XANES spectra for bulk CoFe LDHs and ultrathin CoFe LDHs‐Ar with the corresponding curve‐fitting results; H) the OER performance of bulk CoFe LDHs and the ultrathin CoFe LDH‐Ar nanosheets; I) the corresponding Tafel plots. Reproduced with permission.^73^ Copyright 2017, Wiley‐VCH.

A hydrothermal continuous flow synthesis of NiCo LDH nanosheets as OER catalysts was developed.^84^ First, bulk NiCo LDH nanoplates were synthesized and grown on conductive substrates with a high‐temperature and high‐pressure hydrothermal continuous flow reactor (HCFR) using ammonia and metal salts as a precursors. In this case, Co(III) species were incorporated efficiently into NiCo LDHs because of the easy oxidation of initially formed [Co(II)(NH_3_)_6_]^2+^ to [Co(III)(NH_3_)_6_]^3+^. Meanwhile, the HCSR could also better control the morphology and size of CoNi LDHs due to maintaining a constant precursor concentration in the hydrothermal process. Hence, these synthesized NiCo LDHs grown on carbon paper compared to the traditional hydrothermal method show a thinner thickness and exhibit a better OER activity. Subsequently, the above NiCo LDH nanosheets were partially exfoliated into thinner layers by formamide that led to further improvement of electrocatalytic OER activity. Thus, the exfoliation of NiCo LDH nanosheets leads not only to the exposure of active sites but also initiates a change in the electronic structure of the as‐exfoliated thinner NiCo LDH nanosheets that together promote the enhancement of electrocatalytic activity of NiCo LDHs.^84^ Moreover, the exfoliation process stimulates the surface atoms in the ultrathin 2D sheets to easily escape from the lattice and form a defect structure; meanwhile, the disordered structure can help to lower their surface energy, and hence endow them with better stability.^139^ The structural defects and disorder in the ultrathin 2D sheets can not only reduce the coordination number but also affect the electronic structure, which in turn tunes the activity of the active sites and finally affects the catalytic activities.^73^, ^139^ Recently, numerous studies have demonstrated that the low coordinated Ni, Fe, and Co sites in disordered or amorphous structures especially those are in defects or vacancies positions were considered to be the active sites of electrocatalytic reaction, leading thus to a higher electrocatalytic activity compared to that in perfect structure.^18^, ^72^, ^142^ Therefore, the appropriate insertion of defects to increase the dangling bonds and decrease the coordination number of active sites is considered as an effective strategy to turn the electronic structure of electrocatalyst surface and intrinsically the catalytic activity of active sites. To further confirm the above conclusion, we have recently reported the successful exfoliation of bulk CoFe LDHs into ultrathin CoFe LDH nanosheets having multivacancies by Ar plasma technology, as shown in **Figure** **6**A.^73^ According to scanning electron microscopy (SEM) (Figure 6B) and TEM (Figure 6C), the as‐exfoliated CoFe LDH nanosheets exhibit a monolayer (the thickness of 0.6 nm, Figure 6E) hexagonal structure, indicating that bulk CoFe LDHs are successfully exfoliated to ultrathin CoFe LDH nanosheets. The corresponding (003) and (006) planes are disappeared from XRD, which further proves the exfoliation process (Figure 6D). The as‐exfoliated ultrathin CoFe LDHs show a higher electrocatalytic activity due to increased surface area, more exposed active sites, and edge/corner sites. More importantly, the X‐ray absorption near‐edge structure (XANES) results confirm the formation of multiple vacancies, including O, Co, and Fe vacancies (Figure 6F,G), which further enhances the intrinsically electrocatalytic activity by tuning the surface electronic structure, decreasing the coordination numbers, and increasing the disorder degree of the outstanding surrounding environment of the active sites in ultrathin nanosheets. Thus, the new electrocatalyst exhibits an excellent OER property with lower overpotential of 266 mV and a smaller Tafel slope of 37.85 mV decade^−1^ compared to bulk CoFe LDHs of 321 mV and Tafel slope of 57.05 mV decade^−1^ at the current density of 10 mA cm^−2^ (Figure 6H,I). Therefore, multiple vacancies could be generated conjointly on the OER electrocatalysts to promote both the number of active sites and the intrinsically electrocatalytic activity.

We also prepared an excellent OER electrocatalyst by water plasma, which enables the exfoliation of CoFe LDHs into ultrathin nanosheets and creates multivacancies including oxygen, cobalt, and iron vacancies.^72^ Similarly, this electrocatalyst also exhibits an outstanding OER catalytic activity. Apart from this work, Yang and co‐workers also reported a defect‐rich ultrathin CoFe LDH nanosheets as bifunctional electrocatalysts for overall water splitting.^143^ In this case, CoFe LDHs‐C were prepared by hydrothermal method and CoFe LDHs‐F by exfoliating CoFe LDHs‐C in the mixture of DMF–ethanol. The CoFe LDHs‐F exhibits higher electrocatalytic activity for both OER and HER compared to that of CoFe LDHs‐C. The improvement of both OER and HER properties for CoFe LDHs‐F was attributed to the higher surface area, unsaturated metal active sites with abundant oxygen vacancies, and improved electronic conductivity.

### 3.5 Combining LDH_S_ with Conductive Substrate Hybrids/Composites

As far as we know, bulk LDH‐based electrocatalysts exhibited an inferior electrical conductivity due to intrinsic character of metal hydroxides with weak electrical conductivity. For bulk LDH materials, their poor electronic conductivity has been deemed to a significant factor in limiting their electrocatalytic performance. Moreover, the electronic conductivity of electrocatalysts dramatically affects the kinetic process of OER, resulting in a low electron‐transfer capacity and thus impedes the catalytic reaction, especially the formation of active intermediate —OOH species. To solve these issues, researchers therefore combined the merits of conductive substrates such as nickel foam (NF) and carbon‐based materials (carbon fiber, graphene, carbon nanotube (CNT), and carbon quantum dots) with LDH‐based electrocatalysts to not only enhance the electrical conductivity but also offer higher dispersion to the catalysts, allowing for a large population of metal centers to be electrochemically addressable and accessible.^144^

#### 3.5.1 Porous 3D Nickel Foam

Nickel foam is used as the electrode in alkaline electrolyte due to its high conductive, abundant resource, and porous 3D structure i) to facilitate the dispersion of catalysts to expose more active sites, ii) to increase the contact area between catalyst and electrolyte, and iii) to also accelerate the release of products obtained. For instance, Grätzel and co‐workers synthesized a NiFe LDH grown on NF (denoted as NiFe LDHs/NF) with porous 3D structure.^107^ It exhibits excellent OER properties with low overpotential of only 240 mV at the current density of 10 mA cm^−2^; meanwhile, it shows HER properties with an overpotential of 210 mV at the current density of 10 mA cm^−2^ in 1 m NaOH electrolyte.^107^ Huang et al. also synthesized a Ni_5_Fe LDHs@NF with a 3D hierarchical structure constructed ultrathin nanosheet around outside and the core of alloy around inside. Having this unique structure, the new electrocatalyst exhibits a low overpotential of 210 mV for OER and 133 mV for HER to reach 10 mA cm^−2^ in 1 m KOH, in addition to the low potential of 1.59 V at 10 mA cm^−2^ in 1 m KOH and superior durability when it is used for overall water splitting as a bifunctional electrocatalyst.^145^ Another example was reported by Lu and Zhao, who prepared an OER electrocatalyst by electrodepositing amorphous mesoporous nickel–iron composite nanosheets directly onto macroporous NF substrates.^146^ The as‐prepared oxygen electrode exhibits a high level of catalytic activity toward water oxidation in alkaline solution, which only requires an overpotential of 200 mV to initiate the reaction, and is able of delivering current densities of 500 and 1000 mA cm^−2^ at overpotentials of 240 and 270 mV, respectively. The performance of the NiFe/NF electrodes can be attributed to several factors: i) intrinsically high activity of the NiFe nanosheet catalysts; ii) unique hierarchically porous configuration, which enables the large working surface area and excellent gas bubble dissipation ability; iii) low electrical resistance of the whole water‐splitting cell by using the binder‐free electrodeposition approach and high concentration of electrolytes.^146^

#### 3.5.2 Carbon‐Based Materials

Carbon‐based materials (carbon fiber, graphene, CNTs, carbon quantum dots, etc.) have been extensively used as catalyst supports in water‐splitting system benefiting from their attractively physical and chemical properties mainly related to their high specific area, outstanding thermal stability, high mechanical strength, and good electronic conductivity.^101^ When carbon‐based supports combined with LDH‐based catalyst for water splitting, the electrocatalytic performance is improved significantly and exhibits surprising results.

Recently, Dai and co‐workers developed an OER catalyst by an ultrathin NiFe LDH nanosheet grown on mildly oxidized multiwalled CNTs.^75^ The introduction of multiwalled CNT support could efficiently improve the dispersion and the electronic conductivity of NiFe LDHs that further increase the exposure of active sites and the strong interaction between NiFe LDHs and multiwalled CNTs, accelerating the electron transport in the OER process. Thus, the NiFe‐LDH/CNT catalysts exhibit a high electrocatalytic activity and durability for OER in basic solution and, more importantly, had a high TOF value that was about 3 times higher than those of previously reported for mixed nickel and iron oxide electrocatalyst. Huang and co‐workers also reported an efficient colloidal chemistry strategy for the one‐pot growth of CNT/Fe‐doped Ni LDH nanosheet core–shell superstructures to boost the OER with an ultralow overpotential and high stability.^79^

**Figure 7:**
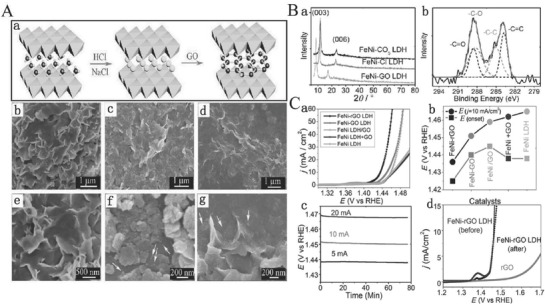
A) Synthesis process of FeNi LDH‐GO by the anion exchange a) and SEM images of as‐synthesized FeNi‐CO_3_ LDHs b,e), FeNi‐Cl LDHs c,f), and FeNi‐GO LDHs d,g); B) XRD pattern and XPS spectra of FeNi LDHs. a) XRD patterns of as‐synthesized FeNi‐CO_3_ LDHs, FeNi‐Cl LDHs, and FeNi‐GO LDHs. b) XPS spectrum of C 1s for as‐synthesized FeNi‐GO LDH hybrid. C) Electrochemical properties of all synthesized catalysts for OER. a) The LSV curves of FeNi LDH, FeNi LDH+GO, FeNi LDH/GO, FeNi‐GO LDH, and FeNi‐rGO LDH on nickel foam electrodes in 1 m KOH electrolyte; b) the corresponding onset potential and potential at a current density of 10 mA cm^−2^; c) chronopotentiometry curves of the FeNi‐rGO LDH on nickel foam at 5, 10, and 20 mA cm^−2^, respectively; d) the LSV curves of FeNi‐rGO LDH and rGO before and after chronopotentiometry measurement at constant a current density of 10 mA cm^−2^ for about 8 h. Reproduced with permission.^76^ Copyright 2014, Wiley‐VCH.

Graphene oxide (GO) nanosheet is an ideal 2D carbon material exhibiting many attractive advantages such as atomic thinness, large surface area, and excellent electrical conductivity after reduction treatment.^147^, ^148^ More importantly, the GO nanosheets with numerous oxygenic groups that could be well assembled with LDH layers carry the positive charge by electrostatic attraction force in aqueous solution, forming charge balancing interlayers in the resulting hybrid sheets.^76^ A typical heteroassembly can be made from graphene and other 2D layers to achieve the full potential of multiple complementing 2D counterparts. This kind of hybrid could show an improved electrocatalytic activity because of its excellent structural features, including highly dispersed and exposed active sites existing in 2D layers, conductive graphene sheets, and strongly synergistic effects between these components.^139^, ^149^ Thus, Yang and co‐workers reported a strongly coupled graphene and FeNi LDH hybrid nanosheets (denoted as FeNi‐GO/rGO LDH) as the electrocatalyst for OER (**Figure** **7**Aa).^76^ SEM is used to characterize the morphology of FeNi LDH at different interlayer anions (Figure 7Ab–g). The X‐ray photoelectron spectroscopy (XPS) and XRD confirm that the GO is reduced and the FeNi‐rGO LDH is prepared (Figure 7B). The OER properties were measured for all the as‐synthesized catalysts. Compared to the original NiFe LDH, the nucleation and growth of NiFe LDH on GO (NiFe LDH/GO), GO or rGO, are hybridized with the interlayer of NiFe LDH (NiFe‐GO LDHs or NiFe‐rGO LDH), which could efficiently promote the OER property. However, there was no change in OER properties in the form of physical mixture of GO and NiFe LDH (NiFe LDH+GO) (Figure 7Ca). In particular, the NiFe‐rGO LDH hybrid compared with all other samples exhibits the most outstanding OER properties since its NiFe exhibits the lowest onset potential (1.425 V) and Tafel slop (39 mV decade^−1^) as well as the smallest overpotential (206 mV) at 10 mA cm^−2^ (Figure 7Cb). Moreover, from all the synthesized samples, the NiFe‐rGO LDH hybrid also has an extremely highest TOF (1 s^−1^) at the overpotential of 300 mV which was also higher than the previously reported LDH‐based catalysts. Furthermore, the NiFe‐rGO LDH hybrid also exhibits a long‐term durability in 1 m KOH electrolyte (Figure 7Cc,d). In this example, the high electrocatalytic activity and properties of NiFe‐rGO LDH could be attributed to the following reasons: i) the intrinsic catalytic activity of NiFe LDH layers; ii) increasing the surface of exposed NiFe hydroxide derived from its unique layered structure; and iii) the interfering of NiFe double hydroxide layer and the rGO, owing to their strong interactions, extremely facilitating the exposure of the catalytically active sites, and enhancing the charge transport through the rGO layers.

**Figure 8:**
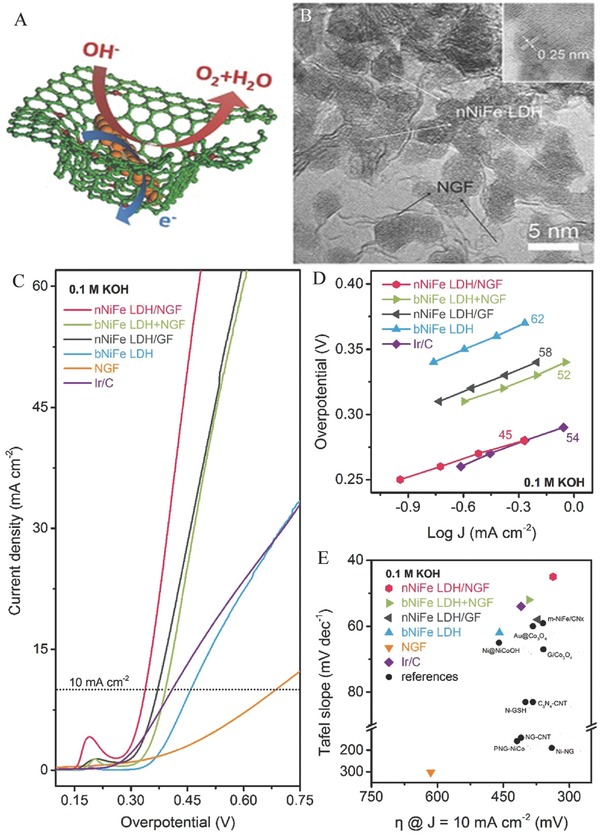
A) Schematic of the spatially confined nNiFe LDH/NGF hybrids. B) Cross‐sectional TEM image of nNiFe LDH/NGF electrocatalyst. C) LSV curves of all samples for OER in 0.1 m KOH electrolyte. D) The corresponding Tafel plots. E) The corresponding kinetics (Tafel slope) and activity (the overpotential required to achieve 10 mA cm^−2^) of all samples in contrast to other references. Reproduced with permission.^78^ Copyright 2015, Wiley‐VCH.

To further stabilize and disperse the transition metals on carbon matrix, according to the electronegativity theory, heteroatom‐doped graphene was more conducive to stabilize and disperse metal atoms or 2D nanolayers on graphene.^150^, ^151^ Thus, Wei and co‐workers reported a novel composite based on NiFe LDHs and graphene using spatially confined hybridization of nanometer‐sized NiFe LDHs into N‐doped graphene frameworks (nNiFe LDH/NGF) (**Figure** **8**A,B).^78^ The N‐doped graphene possess defects to promote the adsorption and anchoring of metal cations, and the mesopores of in‐plane graphene act as nanoreactors to conduct the spatially confined nucleation and growth of nNiFe LDHs, finally leading to a substantial affinity and uniform dispersion of the as‐grown nNiFe LDHs in the mesoporous graphene framework (Figure 8A,B). The as‐synthesized nNiFe LDH/NGF electrocatalyst shows higher OER properties, lower Tafel slope and higher catalytic activity, and exhibits many advantages in the OER process compared to the constant sample and other reported samples (Figure 8C–E).^78^ Zhang and co‐workers also reported a CoAl LDH/3DGN OER electrocatalyst by self‐assembly of single‐layer CoAl LDH nanosheets and 3D graphene network.^77^ The as‐synthesized 3DGN/CoAl‐NS exhibits excellent electrocatalytic activity and durability for OER in alkaline electrolyte, which is comparable or even better than the state of the art of LDH‐related OERelectrocatalysts. The outstanding OER performance and stability shown in this study is presumably profiting from i) the exposure of more active edges that benefit the proton‐coupled electron transfer process in the OER process; ii) large porous structure with high surface area and the electrical interconnection between CoAl LDH nanosheets and conductive 3D graphene network, which not only prevents the single layer CoAl LDH nanosheets from the aggregating but also would increase the accessible surface of the electrolytes and the catalysts; iii) the interaction affinity between single‐layer CoAl LDH nanosheets and 3DGN could accelerate the transfer speed of electron/charge in the OER and improve the reaction kinetics of OER.^77^

**Figure 9:**
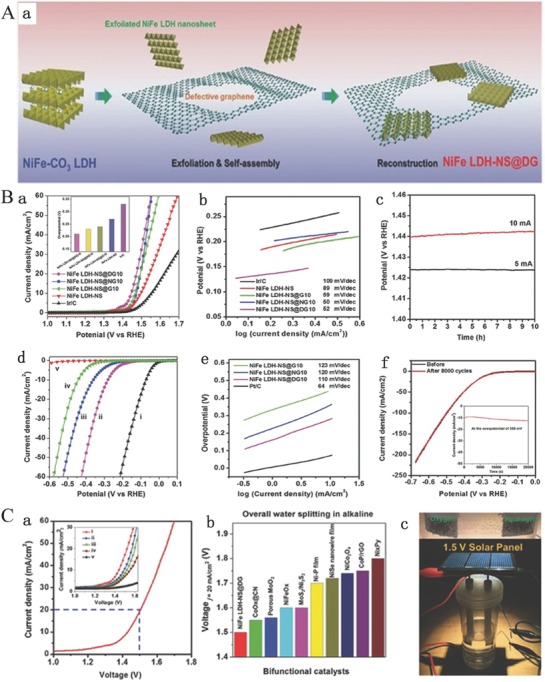
A) Schematic illustration of the fabrication of NiFe LDH‐NS@DG nanocomposite a). B) Electrochemical performance of all synthesized electrocatalysts for OER and HER. a) The LSV curves of all synthesized electrocatalysts for OER in 1 m KOH electrolyte. Inset: The overpotential required at 10 mA cm^−2^. b) The corresponding Tafel slopes for OER. c) Chronopotentiometry curves of the NiFe LDH‐NS@DG10 at constant current densities of 5 and 10 mA cm^−2^, respectively. d) The LSV curves of all synthesized electrocatalysts for HER in 1 m KOH electrolyte. e) The corresponding Tafel slopes for HER. d) The LSV curves for the NiFe LDH‐NS@DG10 before and after 8000 CV cycles. C) The curve of overall water splitting for NiFe LDH‐NS@DG10 on nickel foam with a loading of 2 mg cm^−2^ as bifunctional catalyst in 1 m KOH a). b) To achieve 20 mA cm^−2^, the required voltage for the NiFe LDH‐NS@DG catalyst and other non‐noble metal bifunctional catalysts. c) Demonstration of a solar power–assisted water‐splitting device with a voltage of 1.5 V. Reproduced with permission.^158^ Copyright 2017, Wiley‐VCH.

More importantly, introducing topological defects in graphene has been widely used in the electrocatalytic field owing to a combination of the following reasons. i) The presence of defects in graphene or nanocarbon will efficiently increase the anchor sites that can strongly couple the transitional metal atoms sites by the π–π interaction, finally resulting in fast electron transfer kinetics and excellent stability.^150^, ^152^, ^153^, ^154^, ^155^ ii) Theoretical and experimental results further confirm that certain defect sites in graphene can also be served as the active sites for OER and HER, respectively.^156^, ^157^ Thus, combining defective graphene and LDHs can also serve as a new strategy for the development of electrocatalysts toward the OER process. For example, Yao and co‐workers recently designed a NiFe LDH@DG10 hybrid bifunctional catalyst for water splitting via combining exfoliated NiFe LDH nanosheet and defective graphene, as shown in **Figure** **9**A.^158^ NiFe LDH‐NS, NiFe LDH‐NS@G10, NiFe LDH‐NS@NG, and NiFe LDH‐NS@DG were synthesized and tested for the electrocatalytic performance of both OER and HER, as shown in Figure 9B. The NiFe LDH‐NS@DG hybrid exhibits the most outstanding electrocatalytic activity for OER with an ultralow overpotential of 210 mV at 10 mA cm^−2^ in an alkaline solution among all obtained samples (Figure 9Ba). Moreover, excellent kinetics was shown for reaction with a Tafel slope of 52 mV decade^−1^ (Figure 9Bb). NiFe LDH‐NS@DG hybrid also exhibits higher HER performance with an overpotential of 115 mV at a current density of 20 mA cm^−2^ (Figure 9Bd) and a Tafel slope of 110 mV decade^−1^ (Figure 9Be) in 1 m KOH electrolyte compared with NiFe LDH‐NS. Additionally, this NiFe LDH‐NS@DG hybrid bifunctional catalyst also shows a good durability for OER (Figure 9Bc) and HER (Figure 9Bf) in 1 m KOH electrolyte. This bifunctional catalyst for overall water splitting only needs a voltage of 1.5 V at 20 mA cm^−2^ in 1 m KOH electrolyte that is also superior to other non‐noble metal bifunctional catalysts (Figure 9C). The excellent electrocatalytic performance of NiFe LDH‐NS@DG is ascribed to the following factors: i) defective graphene as a substrate facilitates the constructing of heterostructured composite and shows excellent conductivity and higher specific surface area; ii) the heterostructured composite provides numerous direct interfacial mutual contraction between metal atom and defective graphene which further speeds up the electron transfer and transportation and also narrows the diffusion distance; iii) the highly dispersed NiFe LDH‐NS nanosheets on defective graphene facilitate the exposure of more active sites and provide an ideal model in favor of giving full play to the electrocatalytic advantages of NiFe LDH‐NS; and iv) DFT calculation also confirms that the interaction between NiFe LDHs‐NS and defective graphene plays a synergetic effect on the electrocatalytic activity for OERand HER. Thus, this NiFe LDH‐NS@DG hybrid as electrocatalyst shows outstanding excellent electrocatalytic activity for OER and HER.^158^

In addition to the aforementioned carbon substrates, carbon quantum dots (CQDs) have been considered to be a new kind of nanocarbon with rich functional groups on the surface, which could facilitate the nucleation and anchoring of pristine nanocrystals of CQDs by a strong electrostatic interaction.^159^, ^160^ These kind of CQD materials exhibit many unique physicochemical properties and potential advantages. Notably, the particle size of CQDs is reduced to below 5 nm, and these CQDs show many unique features such as supernal conductivity, rapid electron transfer, and reservoir properties, which could be beneficial for combining LDH‐based materials to improve the electrocatalytic activity of initial LDH‐based materials. Recently, Kang and co‐workers reported CQD/NiFe LDH hybrid electrocatalyst for OER by combining CQDs with a size of about 5 nm and NiFe LDH nanoplates.^161^ This CQD/NiFe LDH hybrid shows a high electrocatalytic activity and stability for OER in 1 m KOH and has an overpotential of 235 mV to obtain 10 mA cm^−2^ in 1 m KOH, which is almost lower than the values of all previously reported NiFe LDH‐based catalysts.

## 4 Advanced LDH Derivatives for Water Splitting

Recently, the derivatives of LDH‐based materials including metal hydroxides, oxyhydroxides, oxides, bimetal nitrides, phosphides, sulfides, and selenides have been widely investigated as electrocatalysts for water splitting. They have exhibited excellent electrocatalytic activities and properties that are mainly attributed to combined advantages of LDHs themselves (the tunability of composition, layered structures, and unique electronic structure) and the unique properties of their derivatives. Because of these merits, the LDH derivative electrocatalysts are endowed to many unique physical and chemical properties and produce the synergistic effect resulting in the significant improvement of electrocatalytic activity and performance.

### 4.1 Metal Hydroxides, Oxyhydroxides, and Oxides

Metal hydroxides, oxyhydroxides, and oxides have been intensively investigated as OER electrocatalysts because of their attractive long‐time stability and activity. Nobel metal oxides, such as RuO_2_ and IrO_2_, have been found to be highly active OER electrocatalysts. However, their high cost, limited resource, and poor stability prevent their scale up. On the other hand, the first‐row 3d transition metal hydroxides, oxyhydroxides, and oxides have exhibited competitive electrocatalytic properties. However, their limited active sites and poor catalytic activities usually lead to the inferior OER property.^12^ LDH‐based derivatives inherit the advantages of LDHs, including a 2D layered structure with large surface area and high exposure of surface atoms, tunability component with unique electronic structure, and intrinsic high active activity.^106^ Therefore, LDH‐based derivatives including metal hydroxides, oxyhydroxides, and oxides as electrocatalysts exhibit an advanced OER property.

**Figure 10:**
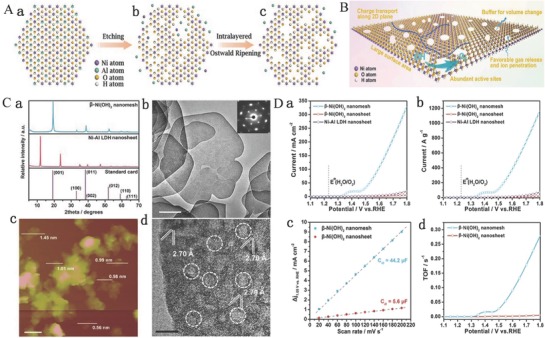
A) The synthesis model of single‐crystalline β‐Ni(OH)_2_ ultrathin nanomeshes. a) The structure of a Ni–Al LDH monolayer. b) The porous β‐Ni(OH)_2_ nanosheets with various pore sizes after etching by strong alkaline solution. c) The formation of single‐crystalline β‐Ni(OH)_2_ ultrathin nanomeshes with abundant and uniform nanopores by the interlayered Ostwald ripening process. B) The structural benefits of the β‐Ni(OH)_2_ ultrathin nanomeshes as OER electrocatalyst. C) Characterization. a) XRD patterns of the single‐crystalline β‐Ni(OH)_2_ ultrathin nanomeshes and as‐exfoliated Ni–Al LDH nanosheets; TEM b), AFM c), and HERTEM d) images of the single‐crystalline β‐Ni(OH)_2_ ultrathin nanomeshes. D) Electrochemical performances of synthesized catalysts in 1 m KOH electrolyte. a) LSV curves of the single‐crystalline β‐Ni(OH)_2_ ultrathin nanomeshes, β‐Ni(OH)_2_ nanosheets, and as‐exfoliated Ni–Al LDH nanosheets. b) The corresponding mass activity. c) The estimation of C_dl_ for β‐Ni(OH)_2_ ultrathin nanomeshes and β‐Ni(OH)_2_ nanosheets. d) TOF plots of β‐Ni(OH)_2_ ultrathin nanomeshes and β‐Ni(OH)_2_ nanosheets at applied potentials. Reproduced with permission.^93^ Copyright 2017, Wiley‐VCH.

Recently, Xie et al. synthesized a single‐crystalline β‐Ni(OH)_2_ ultrathin nanomesh with abundant well‐distributed nanopores using an in situ etching‐interlayered Ostwald ripening process.^93^ First, the exfoliated NiAl LDH ultrathin nanosheets were synthesized (**Figure** **10**Aa). Then, the Al components of the as‐obtained NiAl LDHs were preferentially etched by alkaline solution to form porous β‐Ni(OH)_2_ skeleton (Figure 10Ab). Subsequently, Ostwald ripening confined by the highly anisotropic 2D structure of β‐Ni(OH)_2_ occurred. In this process, the thermodynamically unstable Ni species at protruding sites and edges start to dissolve, and are successively redeposited onto the fringes of large pores to lower the overall energy. Both the dissolution and the redeposition process lead to the shrinking of large pores and finally resulting in the formation of highly porous single‐crystalline β‐Ni(OH)_2_ ultrathin nanomeshes with uniform pore size distribution (Figure 10Ac). This ultrathin nanomesh structure exhibits large surface area and abundant active sites, which extremely facilitates the charge transport, ion penetration, and gas release and buffer for volume change in the OER process (Figure 10B). The XRD patterns confirm the successful synthesis of β‐Ni(OH)_2_ (Figure 10Ca). The TEM (Figure 10Cb), AFM (Figure 10Cc), and high resolution transmission electron microscopy (HRTEM) (Figure 10Cd) also confirm the formation of β‐Ni(OH)_2_ with a structure of highly dense and uniformly distributed ultrathin nanomeshes with the size of 3–4 nm and the thickness ranging from 0.56 to 1.45 nm. The synthesized porous single‐crystalline β‐Ni(OH)_2_ ultrathin nanomeshes shows an outstanding electrocatalytic activity for OER (Figure 10D). At an overpotential of 500 mV, the current density of porous single‐crystalline β‐Ni(OH)_2_ ultrathin nanomeshes reaches 249.4 mA cm^−2^ which was 56.7 times for β‐Ni(OH)_2_ nanosheets, 23.1 times for Ni‐Al LDH nanosheets, and 5.0 times for the previously reported microporous β‐Ni(OH)_2_ nanosheets(Figure 10Da). In addition, it was also found that the single‐crystalline β‐Ni(OH)_2_ ultrathin nanomeshes exhibit higher mass activity (Figure 10Db), expose more active sites compared to the NiAl LDH nanosheets and β‐Ni(OH)_2_ nanosheets, and have electrochemically larger active surface area compared to β‐Ni(OH)_2_ nanosheets (Figure 10Dc). Besides, better durability for OER in 1 m KOH solution was also demonstrated. The outstanding electrochemical activity of β‐Ni(OH)_2_ ultrathin nanomeshes was attributed to i) the structure of ultrathin nanomeshes which exposes more accessible surface area, facilitating the intimate contact with the electrolyte and the diffusion of ion and also accelerating the charge transport in the OER process. ii) The highly dispersed and abundant increased nanopores that create more active phase, especially for those which are adjacent to the nanopores with higher catalytic activity. iii) The numerous nanopores in nanomeshes provide more permeable channels for vertical ion, thus ensuring the electrolyte diffusion into the inside of catalysts. iv) The single‐crystalline nature of β‐Ni(OH)_2_ ultrathin nanomeshes ensures and accelerates the charge transport along the 2D basal planes and finally enhances the electrocatalytic efficiency. v) The highly dispersed and abundant nanopores in β‐Ni(OH)_2_ ultrathin nanomeshes could serve as an extremely effective buffer area in the case of faulty deformation and swelling because of the volume change in a continuous OER process and ensures the electrochemical durability of materials. Jin and co‐workers also reported a porous β‐Ni(OH)_2_ as advanced OER electrocatalysts for OER using a similar method.^86^ First, thin NiGa LDH nanoplates were synthesized as a precursor by a hydrothermal continuous flow method. Subsequently, the Ga^3+^ of NiGa LDH precursor was selectively etched in alkaline solution to form porous β‐Ni(OH)_2_ nanosheets. This porous β‐Ni(OH)_2_ nanosheet as electrocatalysts shows higher OER property and activity compared to NiGa LDH precursor and the one‐step synthesized β‐Ni(OH)_2_ microplates by a hydrothermal continuous flow method.

**Figure 11:**
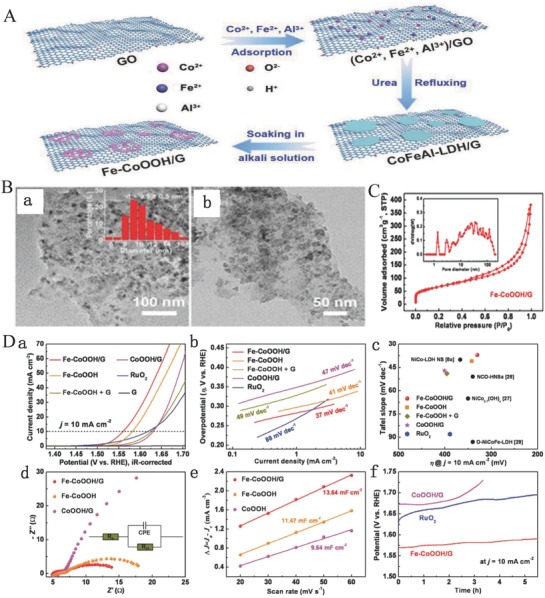
A) Schematic fabrication process for the nanometer‐sized Fe‐CoOOH nanoparticles assembled on graphene by treating the CoFeAl‐LDH/G hybrids in concentrated alkaline solution. B) TEM images of nanometer‐sized Fe‐CoOOH/G nanohybrids a,b). C) N_2_ adsorption/desorption isotherms of the Fe‐CoOOH/G nanohybrids and the pore size distribution calculated from the DFT method. D) Electrochemical performance of all as‐synthesized samples for OER. a) LSV curves; b) the corresponding Tafel plots; c) the corresponding kinetics (Tafel slope) and activity (the overpotential required to achieve 10 mA cm^−2^) of all samples in contrast to other references; d) electrochemical impedance spectroscopy of the as‐synthesized CoOOH/G, Fe‐CoOOH, and Fe‐CoOOH/G electrocatalysts at the potential of 1.60 V; e) the estimation of C_dl_ for CoOOH/G, Fe‐CoOOH, and Fe‐CoOOH/G; f) chronopotentiometry curves of the Fe‐CoOOH/G, CoOOH/G, and RuO_2_ at a constant current density of 10 mA cm^−2^. Reproduced with permission.^92^ Copyright 2017, Wiley‐VCH.

Nanometer‐sized Fe‐modulated CoOOH nanoparticles (Fe‐CoOOH/G) were prepared by an etching process and transformation strategy. The preparation process is described in **Figure** **11**A.^92^ The electrostatic interactions promotes the adsorption of metal ions such as Co^2+^, Fe^2+^, and Al^3+^ to absorb on the surface of GO, which facilitates the growth of CoFeAl‐layered double hydroxide (CoFeAl‐LDH) on the surface of GO sheet (CoFeAl‐LDH/G) under refluxing conditions. Subsequently, the as‐synthesized CoFeAl‐LDH/G was further etched by highly concentrated alkaline solution (12 h) to selectively remove the Al^3+^ in CoFeAl‐LDH/G which results in the formation of porous structure and simultaneously initiates the phase transformation of hydroxide into oxyhydroxide species (Fe‐CoOOH/G). Thus, this specific etching strategy could process hydroxide into oxyhydroxide and form a porous structure (Figure 11B). The Fe‐CoOOH nanoparticles exhibit a nanometer size range from 6 to 16 nm and a thickness of ≈13.31 nm which are dispersed uniformly on the GO sheet surface. The Fe‐CoOOH/G nanohybrids show a high specific surface area of 238 m^2^ g^−1^ and hierarchical pore structure with an average pore size of 9.9 nm (Figure 11C). To confirm the superior OER properties of Fe‐CoOOH/G nanohybrids, comparative samples were also prepared including CoOOH/G, Fe‐CoOOH, FeCoOOH+G, graphene, and RuO_2_. The electrocatalytic performance of all prepared samples demonstrates that the nanometer‐sized Fe‐CoOOH/G nanohybrids exhibit the highest electrochemical activity and performances for OER with only 330 mV overpotential at the current density of 10 mA cm^−2^ and a low Tafel slope (37 mV decade^−1^) in 1 m KOH electrolyte that was much lower than other samples and these properties are comparable to the performances of most reported nonprecious metal‐based materials (Figure 11Da–c). In addition, Fe‐CoOOH/G nanohybrids exhibited a small charge transfer resistance (7.6 Ω), a high electrochemically active area, and good stability (Figure 11De–f). These advanced electrochemical properties could be ascribed to high specific surface area that exposes more active sites and also to the hierarchical pore structure that provides numerous channels for the access and the diffusion of electrolyte ions. In this case, the hybrid constructed from Fe‐CoOOH and graphene produces numerous interface and interaction that mutually accelerates the electron transfer to promote the enhancement of electrochemical properties and activities for OER. Moreover, DFT calculations were also used to calculate the adsorption energy for the intermediates OH, OOH, and O species on the (012) facets to afford much better insight into the high electrochemical activity of nanometer‐sized Fe‐CoOOH/G nanohybrids. The results shows that the incorporation of Fe in nanometer‐sized Fe‐CoOOH particles compared with Co sites assists the improvement of adsorption ability of the active intermediates involved in the OER process. This also explains the excellent OER properties and activities in theory.^92^

Zhang and co‐workers synthesized an ultrafine NiO nanosheet stabilized by TiO_2_ derived from monolayer NiTi LDH precursor,^89^ and NiTi LDH precursors were first prepared by using a reverse microemulsion method that shows a monolayered plate‐like morphology with a diameter of about 20 nm and the thickness of ≈0.8 nm. Then, the as‐synthesized monolayered NiTi LDH nanosheets were calcined to form NiTi mixed oxide nanosheets at 500 °C (denoted as Mono‐NiTi‐MMO). The Mono‐NiTi‐MMO nanosheets exhibit a particle size of ≈20 nm and an atomic thickness. Their studies unveil that the NiO mainly exposed (110) facets with a likely hexagonal geometry that has a higher surface energy and surface chemistry reactivity. The anatase TiO_2_ with dominant (001) facets could make the NiO nanosheets highly dispersed, avoiding the aggregation of NiO nanosheets, leading to the formation of NiO/TiO_2_ heterostructure. For the sake of comparison, Bulk‐NiTi‐MMO with particle size and thickness of ≈100 and ≈13 nm, respectively, was synthesized and also compared with the commercial NiO nanosheets with a particle size of ≈25 nm. As a result, the Mono‐NiTi‐MMO nanosheets exhibit higher average Ni oxidation state. After investigating the coordination environment surrounding the Ni atoms in Bulk‐NiTi‐MMO, the commercial NiO, and the Mono‐NiTi‐MMO nanosheets, respectively, the first Ni–O shell with a coordination number (*N*) of 5.70 for Mono‐NiTi‐MMO has a distance of ≈2.05 Å that was lower than 2.08 Å found in both NiO and Bulk‐NiTi‐MMO. Moreover, the *N* of the Ni–Ni shell for Mono‐NiTi‐MMO also found to be only 7.70 that is apparently lower than 12.00 for both NiO and Bulk‐NiTi‐MMO, confirming the formation of nickel vacancies (V_Ni_) in the as‐obtained Mono‐NiTi‐MMO nanosheets after calcination of the precursor. Moreover, the distance of Ni–Ni shell found to be ≈2.98 Å for Mono‐NiTi‐MMO, which is larger than that of the contrast samples of 2.96 Å and also demonstrates the presence of Ni^3+^, Ti^3+^, and oxygen vacancies in Mono‐NiTi‐MMO nanosheets, which endows high electrical conductivity to the Mono‐NiTi‐MMO and further accelerated the charge transfer of the process for water splitting. The electrocatalytic performance shows that the Mono‐NiTi‐MMO exhibits the most excellent electrocatalytic performance reaching 10 mA cm^−2^ at an overpotential of 320 mV which was about 3, 6, 15, and 34 times corresponding to the Mono‐NiTi LDH precursor, NS‐NiO, NiO, and Bulk‐NiTi‐MMO, respectively. Also, the Mono‐NiTi‐MMO shows the smallest Tafel slope and charge transfer resistance in contrast to other samples. The Mono‐NiTi‐MMO also has a good stability in 1 m KOH. DFT calculation also demonstrates that the introduction of V_Ni_ and oxygen vacancies (V_O_) for Mono‐NiTi‐MMO improves the carrier concentration and the electrical conductivity because of the remarkably increased density of states (DOS) near the Fermi level of NiO‐V_Ni_/TiO_2_‐V_O_. Mono‐NiTi‐MMO (denoted as NiO‐V_Ni_/TiO_2_‐V_O_) also has a larger adsorption energy for H_2_O than other samples (defect‐free NiO and TiO_2_, NiO‐V_Ni_, and NiO‐V_Ni_/TiO_2_). The charge distribution of the orbital wave function at the valence band maximum of NiO‐V_Ni_ shows that the charge density of the defective NiO mainly concentrates at the O atoms near V_Ni_. All the above factors including ultrafine and ultrathin nanosheet structure, high surface reactivity of exposing (110) facets, high proportion of Ni^3+^ and Ti^3+^, unique electronic structure of NiO‐V_Ni_/TiO_2_‐V_O_ including V_Ni_ and V_O_, and the optimum adsorption of H_2_O are all jointly responsible for the excellent OER property of Mono‐NiTi‐MMO nanosheets.

Hu and co‐workers also reported a unique in‐grown structure of NiFe‐MMO/CNT hybrid OER catalyst by calcination of the NiFe LDH/CNT precursor that also exhibits excellent OER property compared with the NiFe LDH/CNT precursor.^87^ The aforementioned works confirm that the morphology structure, components, surface reactivity, and the surface electronic structure of the as‐obtained metal oxides by LDH‐based materials as precursors could be efficiently tuned to improve their electrocatalytic performance.

### 4.2 Bimetal Nitride/Phosphide Electrocatalysts

Recently, transition metal phosphides (TMPs) and nitrides have been widely investigated owing to their many desired features such as high corrosion resistance and electrical conductivity. Both metal phosphides and nitrides have a metallic nature, which can promote the electron transfer during the catalytic oxidation and reduction of water. Since they have been reported as OER or HER electrocatalysts, many researchers have developed various transitional metal phosphides and nitrides with special structures or specific compositions. Many of them show excellent electrochemical performance and great potential for the commercial application of water splitting. With so many eye‐catching findings, some researchers have claimed that both bimetal phosphides and nitrides have much better electrochemical performance than of the pure one, probably due to the synergistic effect. As far as we know, LDHs are a class of 2D materials, where a fraction of divalent metal ions coordinate octahedrally by hydroxyl groups in the brucite‐like layers that are uniformly replaced by trivalent metals with the molar ratio of M3+/(M3++M2+). Therefore, LDHs can be selected as a good precursor to prepare bimetal phosphides or nitrides. The prepared bimetal phosphides or nitrides can still maintain their layered structure, which may help them to expose more active sites and bring excellent performance. Thus, more attention should be paid to the LDH precursor, which may be considered as an ideal intermediate for the preparation of various bimetal phosphides or nitrides. Therefore, in the following section, we will review various bimetal phosphides or nitrides derived from LDHs that have been recently reported and can help the readers to know more about this field. Depending on their active element, bimetal phosphides and nitrides would be discussed separately.

TMPs are an important kind of compounds that are formed by the alloying of metals and phosphorus, which can bring dramatic effect on their electronic performance. Since the first report on the implementation of TMPs as electrocatalysts for water splitting, many researchers have paid their attention to the development of various TMP materials as HER or OER electrocatalysts.^162^, ^163^, ^164^ Both the structure and the composition could be constructed and controlled to enhance the electrocatalytic activities.^165^, ^166^ Taking into consideration that the incorporation of another metal element in TMPs could significantly improve their electrochemical performance, LDHs would be a good candidate to drive various TMPs for their layer structure and the composition of different metals. The extra metal integrated into the TMPs could change their electronic structure and increase their activities and stabilities. For example, Sun and co‐workers reported that the low‐temperature phosphidation reaction results in the formation of Fe‐doped CoP nanoarray from the CoFe hydroxide precursor.^132^ For the doping effect, the Fe‐doped CoP showed much better electrochemical performance than that of CoP. And the prepared electrode only needs an OER overpotential 230 mV and an HER overpotential of 78 mV for 10 mA cm^−2^ in 1.0 m KOH, with the need for a cell voltage of 1.60 V for 10 mA cm^−2^ water‐splitting current in the two‐electrode electrolyzer, which is superior to most of the reported non‐noble metal catalysts. Moreover, for the easy adjustability of LDH materials, this kind of precursor can be employed to study the real effect of extra metal in TMPs by changing the amount ratio. For instance, Hu and co‐workers have reported that the bimetallic (Fe*_x_*Ni_1−_ *_x_*)_2_P nanoarrays act as exceptionally efficient electrocatalysts for OER in alkaline and neutral media.^167^ Both the shifted XRD and XPS results showed that the extra Fe in Ni_2_P could modulate the electronic structure by introducing Fe into the Ni_2_P lattice. The difference could increase the local electric dipole, which can make it easier for the adsorption and desorption of OER reactants and products, and lead to a lower kinetic barrier and higher catalytic activity. In considering these advantages, embedding extra metal makes the catalyst excellent in OER, and only needs a low overpotential of 156 and 255 mV to reach 10 and 500 mA cm^−2^ current densities, respectively, in 1 m KOH. It also shows a good activity in a 0.1 m phosphate buffer, with only small overpotential of 396 mV to reach a current density of 10 mA cm^−2^. Currently, with many published reports, people have understood these bimetal phosphide systems more and more clearly and can further improve their performance.

**Figure 12:**
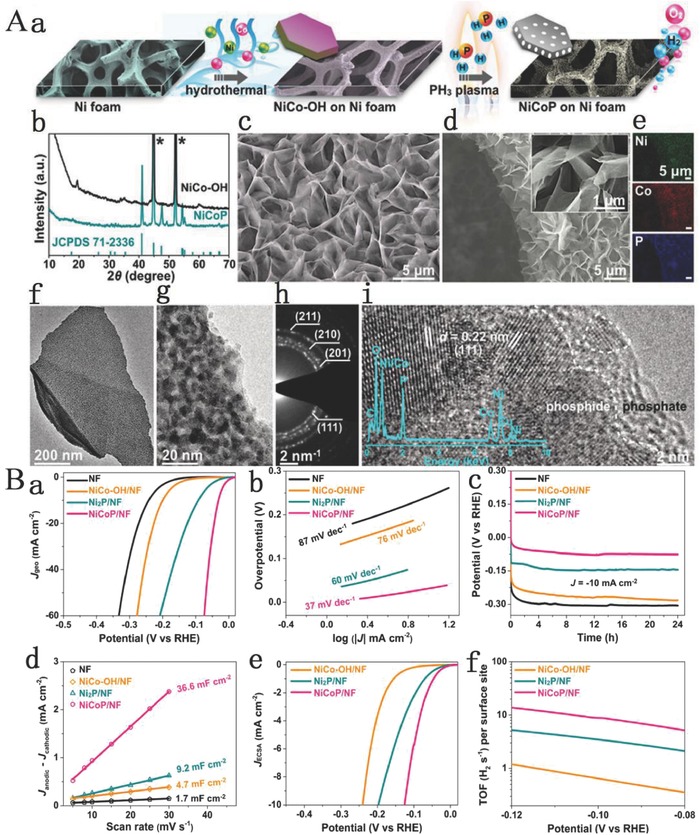
A) Synthetic route and structural characterization of the NiCoP nanostructure. a) Schematic illustration of the synthetic route for NiCoP nanostructure on Ni foam. b) XRD patterns of NiCo‐OH and the converted NiCoP. The asterisks mark the diffraction peaks from Ni foam. c) SEM image of NiCo‐OH. d) SEM images and e) the corresponding EDS elemental maps of the NiCoP. f,g) TEM images, h) SAED pattern, and i) high‐resolution TEM image and EDS spectrum (inset) of the NiCoP. The dashed white line highlights the crystalline–amorphous boundary. B) HER electrocatalysis in 1 m KOH. a) IR‐corrected polarization curves per geometric area of the NiCoP/NF recorded at a scan rate of 3 mV s^−1^, along with Ni_2_P/NF, NiCo−OH/NF, and NF for comparison. b) Polarization curve–derived Tafel slopes for the corresponding electrocatalysts. C) OER electrocatalysis in 1 m KOH. a) IR‐corrected polarization curves per geometric area of the NiCo‐P/NF recorded at a low scan rate of 0.5 mV s^−1^, along with Ni−P/NF, NiCo−OH/NF, and NF for comparison. b) Polarization curve–derived Tafel slopes for the corresponding electrocatalysts. D) NiCoP/NF electrocatalyst for overall water splitting in 1 m KOH. a) Schematic illustration of two‐electrode cell using NiCoP/ NF for both anode and cathode for water splitting. b) Polarization curve recorded at 0.5 mV s^−1^. Inset: Digital photograph of the two electrode configuration. c) Long‐term stability test carried out at constant current densities of 10, 20, and 50 mA cm^−2^. Reproduced with permission.^94^ Copyright 2016, American Chemistry Society.

As far as we know, the creation of various defects in nanomaterials would have a great effect on their chemical and physical properties, which may change their electrochemical performance. Some published reviews also focused on the positive effect of defects in the field of electrocatalysis.^168^ Therefore, considering the effect of defects on the bimetal phosphide, we were wondering whether the defects of the bimetal phosphide would further enhance their OER or HER performance. As expected, Alshareef and co‐workers recently reported a novel PH_3_ plasma‐assisted approach to convert NiCo LDHs into ternary NiCoP as a novel bifunctional electrocatalyst (**Figure** **12**Aa).^94^ The plasma technology can etch the surface of nanomaterials and bring different defects.^168^ Toward this goal, we recently reported many interesting works by using this useful technology, for example, by inducing the oxygen vacancy in Co_3_O_4_, the OER activity is improved.^28^ Here, the plasma technology was employed for the preparation of NiCoP, and found not only to save time and energy but also induce some defects in the materials.^94^ The author used DFT calculations to study how the Co substitution affects the electronic structure of Ni_2_P and the surface adsorption energy of the reactants and thus the electrocatalytic activity. Both Ni_2_P and NiCoP showed no bandgap, and metallic nature, which would promote the electron transfer. Moreover, the Gibbs free energy of the adsorbed H* was used to evaluate the HER performance. As we can see, the NiCoP has a better HER performance than that of Ni_2_P. Surprisingly, from STEM results, we can see that lattice defects were obviously created by this plasma technology (Figure 12A). These advantages synergistically contributed to the high OER and HER performance. It only takes a low overpotential of 32 mV at 10 mA cm^−2^ in alkaline media for HER. Moreover, a current density of 10 mA cm^−2^ is achieved at an overpotential of 280 mV for OER (Figure 12 B,C). Both the HER and OER overpotentials are among the best‐published values for non‐noble metal catalysts. Furthermore, it only requires a cell voltage of 1.58 V to reach a current density of 10 mA cm^−2^, showing the most efficient electrocatalysts for water splitting (Figure 12D). Although the bimetal phosphides derived from LDHs have important implications or roles in water splitting, there is still some unknown space to be discovered by researchers.

Transition metal nitrides (TMNs) are very similar to TMPs and also have a metallic nature, which can certainly promote and faster the electron transfer during the electrocatalytic reaction. Since Wu and co‐workers first reported the Ni_3_N nanosheets with excellent OER catalytic activity, new avenues for the study of various TMNs have been opened up.^62^ In broad terms, one might expect that the bimetal may also enhance the performance of TMNs for OER and/or HER. The extra metal introduced in TMNs can optimize both their valence and electronic states, leading to enhanced electrocatalytic performance. Therefore, LDHs can be chosen as an ideal platform to prepare various TMNs and help study the bimetal nitrides as water‐splitting catalysts. Different kinds of bimetal nitrides and their application in water splitting have been investigated. For instance, Ni_3_FeN nanoparticles (Ni_3_FeN‐NPs) with a particle size of 100 nm and a thickness of about 9 nm were successfully fabricated by thermal ammonolysis of ultrathin Ni_3_Fe‐LDH nanosheets.^169^ The bimetal Ni_3_FeN‐NPs show the best HER activity compared to that of Ni_3_N, Ni_3_FeN‐bulk and NiFe‐metal oxide, and only an overpotential of 158 and 416 mV is needed to reach a current density of 10 and 200 mA cm^−2^, respectively, which is even better than the commercial Pt/C. In addition, the more positive onset potential and lower Tafel slope further confirm that Ni_3_FeN‐NPs are ideal HER electrocatalysts. The bimetal Ni_3_FeN‐NPs also show the best OER performance, which only takes overpotential of 280 mV at the current density of 10 mA cm^−2^. DFT calculations were taken to understand the origin of the excellent activity of Ni_3_FeN for both HER and OER. From the band structure and DOS, we can see that Ni_3_FeN are continuous near the Fermi level with no obvious gap, indicating that Ni_3_FeN is metallic, which ensures fast charge transport and well electrical conductivity that are very important in electrocatalytic reactions. The calculation of the adsorption behavior of H_2_O is also calculated on the surfaces of these metal nitrides and oxide surface and shows that the special electronic structure of the binary metal nitrides promotes the adsorption of H_2_O. So, the bimetal nitrides can be used as ideal bifunctional water‐splitting electrocatalysts. To further improve the activity of bimetal nitrides, they can be combined to various 3D supports to avoid using any binder, which may have a negative effect on both activity and stability. Direct growth of NiFe bimetal nitrides on a substrate such as Ni foam could effectively solve this problem. For example, Tang and co‐workers have successfully prepared NiFe bimetal nitride nanostructures on the surface‐redox‐etched Ni foam (FeNi_3_N/NF) as a highly efficient bifunctional electrocatalyst for overall water splitting.^98^ Benefiting from the advantages of the in situ unique electrode fabrication, bimetallic composite, and metallic nitrides, the FeNi_3_N/NF electrocatalysts exhibit excellent performance for both HER and OER, which only require low overpotential of 75 and 202 mV at 10 mA cm^−2^, and Tafel slopes of 98 and 40 mV decade^−1^, respectively. This in situ preparation method could also endow the catalyst with excellent stability and suitable properties for application in water splitting, which can work more than 400 h of consistent galvanostatic electrolysis without any visible voltage elevation. It was also found that the existing defects in the TMNs can have a great effect on their chemical and physical properties. Therefore, various methods were developed to induce different kinds of defects to improve their performance. Recently, we reported some exciting results about the generation of defects on the bimetal nitrides. For instance, nanoparticle‐stacked porous Ni_3_FeN (NSP‐Ni_3_FeN) electrocatalyst can be used as a bifunctional catalyst for both HER and OER.^90^, ^91^ From SEM images, we can see that the Ni_3_FeN can keep the LDH layer structure, and the nanosheets are composed of stacked nanoparticles, exposing more active sites for electrocatalytic reactions. Interestingly, the HRTEM results show the existence of much boundaries, defects, and dislocations, which were widely considered to be active sites for electrocatalysis. Taking these advantages together, the catalyst exhibits excellent OER performance, which only requires a low overpotential of 223 mV to reach a current density of 10 mA cm^−2^ and HER property with a very low overpotential of 45 mV.

Although many of these works have been reported, there is still a lot to be explored. For example, it is highly important to investigate the effect of both defects of metal atoms and nitrogen atoms on the activity. The changes of defects during the OER or HER processes should also be studied. Therefore, much more work is required to make the above problems clearly and we believe that more exciting findings would be reported shortly.

### 4.3 Bimetal Sulfides/Selenides

Transition metal dichalcogenides (TMDs) have been widely researched as electrocatalysts for HER or OER, especially MoS_2_.^170^, ^171^, ^172^ MoS_2_ has attracted considerable attention since its invention as an HER electrocatalyst. It was found that the edge sites were more active than the one at the basal plane. Having this in mind, various strategies have been developed and implemented to improve the activity of MoS_2_ by exposing more edge sites. On the other hand, some strategies have also been described to activate the basal plane by doping other metal atom and so on.^173^, ^174^ Moreover, the activity of MoS_2_ was not good enough to meet the demand of large production. So, some researchers focused their attention on other transition metal dichalcogenides (Fe, Co, and Ni). It has been reported that a certain amount of Fe doping could significantly improve the OER performance of nickel hydroxide/oxide by adjusting their electronic structure. Therefore, it is expected that doping of a certain amount of Fe to nickel sulfides could also enhance the catalytic activity of HER.

**Figure 13:**
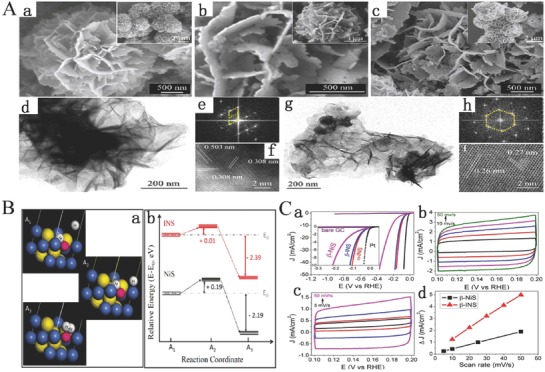
A) SEM images of a) FeNi LDH precursor, b) β‐INS nanosheets, and c) metallic α‐INS nanosheets. TEM images d,g), HRTEM images f,i) and the corresponding Fourier transformed patterns e, h) of the β‐INS d–f) and the α‐INS g–i) nanosheets. B) Schematic reaction pathway of HER on α‐INS ultrathin nanosheets in acid environment a). b) Kinetic energy barrier profiles of HER on α‐INS and α‐NiS nanosheets. The yellow, blue, and red spheres in A1−A3 represent S, Ni, and Fe atoms, respectively. C) ECSA tests of catalysts toward HER in the acidic electrolytes of 0.5 m H_2_SO_4_. a) LSV curves of β‐NiS‐, β‐INS‐, and α‐INS‐catalyzed HER, and b) CV curves of β‐INS and c) β‐NiS nanosheets with various scan rates. d) Charging current density differences plotted against scan rates. The linear slope, equivalent to twice the double‐layer capacitance, *C*_dl_, was used to represent the ECSA. Reproduced with permission.^95^ Copyright 2015, American Chemistry Society.

Recently, Yang and co‐workers have successfully prepared iron–nickel sulfide (INS) ultrathin nanosheets as highly efficient HER electrocatalysts, which showed much batter activity and stability than that of nickel sulfide.^95^ The authors also discussed the activity of INS with different phases (α, β). While the β‐INS ultrathin nanosheets were prepared by a topotactic conversion reaction, derived from FeNi LDH nanosheets (as shown in **Figure** **13**), the metallic α‐INS nanosheets were obtained by annealing treatment. The electrochemical measurement indicates that the metallic α‐INS nanosheets show the best HER activity with an overpotential of 105 mV at 10 mA cm^−2^ and a smallest Tafel slope of 40 mV decade^−1^ among the compared samples (Figure 13C). To further understand the origin of this highly interesting activity, the DFT calculation was undertaken to compare the detailed HER pathways on both α‐NiS and α‐INS (Figure 13B), which shows that the Fe incorporation could change the electronic structure of the catalytically active center and further facilitate the HER process. To further investigate the energy profiles through the HER process on α‐INS and α‐NiS, the energy barrier of different step is also carried out and shows that the energy barrier for the rate‐determining step (the combination of H_ad_ with H^+^) is only 0.01 eV on the α‐INS, which was much lower than that on NiS (0.19 eV). The released energy for the formation of H_2_ on α‐INS (2.39 eV) was also more significant than that on NiS (2.19 eV). Therefore, the bimetal sulfide showed much better HER activity than the monometal sulfide for its lower energy barrier for H^+^ adsorption and higher exothermicity for the formation of H_2_. This work could initiate other investigation for the application of various TMDs to the electrocatalytic fields. Recently, some sulfide can also be used successfully as the electrocatalysts of the OER process and even be employed as bifunctional catalysts.^175^ As discussed in the aforementioned cases, the bimetal sulfides also show a better OER performance than that of monometal. Therefore, LDHs have been identified as precursors for the preparation of layered bimetal sulfides for OER electrocatalysts. With many excellent achievements in this field, TMDs were demonstrated to be oxidized during the OER process, which would result in the formation of oxides or hydroxides on their surface.^176^ This drives us wonder about the real active sites of TMDs for OER. Thus, some people compared the TEM images of TMDs before and after the long‐time OER process. Most of the results showed that a thin oxide or hydroxide would be formed on the top surface of the TMDs, which may react as the real active sites for the OER.

Recently, nickel selenide has been reported to be entirely converted into nickel hydroxide under oxygen‐evolution conditions,^96^ which shows that the metal selenides were unstable during oxygen evolution condition. This has inspired them to adapt new methodology based on using metal selenides as templating precursors for the preparation of highly active metal oxide OER catalysts. The bimetal selenides derived from LDHs were used as templating precursors and then transformed in situ into oxides, which catalyze OER with an overpotential of only 195 mV for a current density of 10 mA cm^−2^. Although there exist many reported papers about the efficiency of TMDs in OER electrocatalysts, we should understand that the real active sites were still the surface oxides or hydroxides. Moreover, the difference between the oxides transformed from the TMDs with those prepared directly should be further investigated.

## 5 Summary and Outlook

Electrolyzing water is considered to be one of the most effective ways to generate hydrogen. OER and HER are two critical half reactions of water splitting which dramatically affect the efficiency of hydrogen production. Thus, designing and fabricating highly active and stable electrocatalysts are essential to improve the efficiency of generating hydrogen. Currently, 3d transition‐metal LDH‐based materials and their derivatives (metal hydroxides, oxyhydroxides, oxides, bimetal nitrides, phosphides, sulfides, and selenides) as electrocatalysts for OER and HER have attracted much attention benefiting from their tunable 2D lamellar structure, various component, and changeable intrinsic electronic structure. A limited number of active sites and their poor intrinsic activity are deemed the most important two factors affecting the electrocatalytic active of LDH‐based materials and limiting their practical application. In this review, we summarized and concluded the strategies to improve the electrocatalytic activity of LDH‐based electrocatalysts for OER and HER. We consider the following as defining the main strategies to achieve these objectives.

i) Morphology control: The morphology of multilayer stacked nanosheets for bulk LDH materials is hugely detrimental to the exposure of active sites, the diffusion of reactants, and the releasing of resultants in the OER process. The morphology also results in a reduced electronic conductivity and inferior intrinsic catalytic activity of active sites. Therefore, synthesizing and fabricating LDH catalysts with ultrathin nanosheets or hierarchical nanostructure can increase the specific surface area and the number of electrocatalytic active sites, which can promote the electrolyte penetration and the oxygen or hydrogen bubbles release, accelerate the charge transport and transfer, improve the electrical conductivity and the intrinsic catalytic activity, and finally enhance the electrocatalytic performance.
ii) Cationic doping: Cationic doping has also been considered as part of an efficient and alternative strategy for promoting the improvement of electrocatalytic property for LDH‐based electrocatalysts. Cationic doping may change the morphology (the thickness and size), which can enhance the electrical conductivity and improve the kinetic process. Moreover, doping atom can also serve as the active sites regulating the surface electronic structure of electrocatalyst and enhancing the intrinsic catalytic activity of LDH‐based electrocatalysts. Thus, cations doped in LDH‐based electrocatalysts for water splitting can further regulate and improve the catalytic performance.
iii) Tuning the anion and spacing in the interlayer: Unique atomic distribution structure endows LDHs with unique flexibility to regulate the interlayer distance and the types of intercalation anion in the interlayer region. Moreover, increasing the interlayer distance of intercalation anion could expand the accessible surface areas of LDH electrocatalysts and efficiently facilitate the penetration and diffusion of electrolyte. Meanwhile, these kinds of intercalated anions could also regulate the surface electronic structure at the surrounding of active atoms, resulting in the improvement of the intrinsic catalytic activity of active sites. Thus, monitoring and controlling the interlayered anion in LDH‐based materials could promote the enhancement of their electrocatalytic properties and activities.
iv) Exfoliation of LDH/tuning electronic structure and introducing defects: Compared to the bulk LDHs, the as‐exfoliated single or few atomic layer LDH nanosheets exhibit a higher surface area, a better electrical conductivity, and intrinsic electrocatalytic activity, leading to a higher electrocatalytic performance. Furthermore, the exfoliation of LDHs is accompanied with the formation of edge, corner, and defect sites with higher catalytic activity. Moreover, introducing additional proper defects by plasma technology in the as‐exfoliated single or few atomic layer LDH nanosheets results in the formation of numerous edge and corner sites, produces more dangling bonds, and decrease the coordination number of the active sites. Meanwhile, the existence of defects such as oxygen vacancies and metal vacancies could regulate and control the electronic structure at the surrounding of active sites that are adjacent to defect sites, which improves the intrinsically catalytic activity of active sites and the adsorption of intermediate species in the OER process. Thus, exfoliating LDH into ultrathin LDHs and simultaneously introducing defects are extremely advantageous strategies, producing a synergistic effect that efficiently improves the OER activities and properties of LDH‐based electrocatalysts.
v) Combined with conductive substrate hybrids/composites: The poor conductivity and the aggregation of LDH nanosheets usually lead to a slow electron transfer capacity, limit the full contract between the electrolyte and electrocatalysts, and also suppress the catalytic activity of active sites, which all hinder their large‐scale application. On the other hand, conductive substrates such as nickel foam and carbon‐based materials (carbon fiber, graphene, CNT, carbon quantum dot, etc.) that are known to exhibit many advantages, such as excellent electrical conductivity, favoring the high dispersion of catalyst, exposing a large population of metal centers (active sites) to be electrochemically addressable and accessible, could contribute to a fraction of electrocatalytic performance. More importantly, the formation of hybrids/composites between LDHs and conductive substrates could accelerate the electron transfer, shorten the diffusion distance, and also contribute to strong synergistic effect in the OER process. Therefore, combining the merits of both LDHs and conductive substrates could significantly enhance the electrocatalytic performance of electrocatalysts.
vi) Processing of the LDH derivatives: The derivatives of LDH‐based materials such as metal hydroxides, oxyhydroxides, oxides, bimetal nitrides, phosphides, sulfides, and selenides as electrocatalysts have exhibited excellent electrocatalytic activity and many advantages are lacking in LDH‐based materials. For example, while metal nitrides and phosphides exhibit corrosion resistance and electrical conductivity, sulfides and selenides possess long‐term stability in acidic medium. LDH derivatives could also inherit outstanding features such as the tunability of composition, and layered and unique electronic structures. Therefore, combining these advantages makes the LDH derivatives to show preeminent electrocatalytic activity and performance.

According to our summary, there is no doubt that LDHs and their derivatives are considered to be the most promising electrocatalysts for water splitting. In particular, the shortcomings of LDH electrocatalysts have been remedied using various strategies to improve their electrocatalytic performance. Overall, growing LDH electrocatalyst on conductive substrates with a 3D hierarchical and open nanostructure leads to the formation of new performant composite possessing further properties not existing in the individual components, such as the creation of more defect sites that will facilitate the production of highly efficient electrocatalytic activity with ultralow overpotential, long‐term operational stability, and especially make the larger current density to be close to the current application. Therefore, it is interesting to anticipate that the preparation of new composite‐type LDH electrocatalyst for water splitting will be the future trend. Apart from, LDHs and their derivatives will also be extended to other fields such as the fabrication of light emitting devices, battery materials, supercapacitors, and flame‐retardant nanocomposites.

## Conflict of Interest

The authors declare no conflict of interest.
